# Outcomes, prognostic factors, and the role of intracranial pressure monitoring in severe community-acquired bacterial meningitis: a multicenter retrospective cohort study

**DOI:** 10.1016/j.lanepe.2026.101767

**Published:** 2026-07-06

**Authors:** Cindy Poirier, Nicolas Terzi, Nicolas Roesch, Laurent Argaud, Sarah Jouin, Marc Le Pape, Jean-Rémi Lavillegrand, Hervé Hyvernat, Romain Tymen, Pierrick Lafarge, David Bougon, Pierrick Bauduin, Jérémie Mallet, Julien Jabot, Guillaume Navarro, François Arrivé, Juliette Bernier, Michel Badet, Maxens Decavele, Anaïs Curtiaud, Eddine Bendiab, Baptiste Balanca, Benoit Champigneulle, Sami Hraiech, Loïc Le Guennec, Elodie Fournier, Emmanuel Canet, Romain Sonneville, Guillaume Dumas, MAC-ICU Study Group, François Beloncle, François Barbier, Nicolas de Prost, Mathieu Jozwiak, Cécile Aubron, Frédéric Pène, Damien Du Cheyron, Tai Pham, Phan Hoang, Florian Contard, Clément Brault, Rémi Coudroy, Hafid Ait Oufella, Ferhat Meziani, Kada Klouche

**Affiliations:** a Service de Médecine Intensive-réanimation, CHU Grenoble-Alpes, Univ Grenoble Alpes, HP2 Laboratory, Inserm U-1300, France; b Service de Médecine Intensive-réanimation, CHU de Rennes, Université de Rennes, France; c Service de Médecine Intensive-réanimation, CHU de Nantes, Université de Nantes, France; d Service de Médecine Intensive-Réanimation, Hôpital Édouard Herriot, Hospices Civils de Lyon, Université Lyon 1, France; e Service de Médecine Intensive-Réanimation, CHU Angers, Université d’Angers, France; f Service de Médecine Intensive-Réanimation, CHU d’Orléans, Orléans, France; g Service de Médecine Intensive-réanimation, Hôpitaux Universitaires Henri-Mondor, Créteil-AP-HP, France; h Service de Médecine Intensive-Réanimation, Centre Hospitalier Universitaire l'Archet 1, Université Côte d’Azur, Nice, France; i Service de Médecine Intensive-Réanimation, CHRU de Brest, Université de Bretagne Occidentale, Brest, France; j Medical Intensive Care Unit, Cochin Hospital, Hôpitaux Universitaires Paris Centre, Paris-AP-HP, Université Paris Cité, Paris, France; k Service de Réanimation Polyvalente, Centre Hospitalier Annecy Genevois, Annecy, France; l Service de Médecine Intensive-Réanimation, CHU Caen, Université Caen Normandie, Caen, France; m Service de Médecine Intensive-Réanimation, Hôpital de Bicêtre-AP-HP, Hôpitaux Universitaires Paris-Saclay, Université Paris-Saclay, Paris, France; n Service de Réanimation Polyvalente, CHU de La Réunion, Site Nord, Université de la Réunion, Saint-Denis, France; o Service de Médecine Intensive-Réanimation, CHU Amiens-Picardie, Université de Picardie Jules Verne, Amiens, France; p Service de Médecine Intensive-Réanimation, CHU Poitiers, Université de Poitiers, Poitiers, France; q Service de Médecine Intensive-Réanimation, Hôpital Saint-Antoine, Paris- AP-HP, Sorbonne-Université, Paris, France; r Service de Réanimation Polyvalente, Centre Hospitalier Métropole Savoie, Chambéry, France; s Service de Médecine Intensive - Réanimation, Département R3S, Groupe Hospitalier Universitaire AP-HP-Sorbonne Université, Paris, France; t Service de Médecine Intensive-Réanimation, Nouvel Hôpital Civil de Strasbourg, Hospices Civils de Strasbourg, Université de Strasbourg, Strasbourg, France; u Service de Médecine Intensive-Réanimation, Hôpital Lapeyronie, CHRU Montpellier, Université Montpellier 1, Montpellier, France; v Neurological Anesthesiology and Intensive Care Unit, Hôpital Pierre Wertheimer, Hospices Civils de Lyon, Université Lyon 1, Lyon, France; w Service de Réanimation Neurochirurgicale, Univ. Grenoble Alpes, Inserm, CHU Grenoble Alpes, HP2, Grenoble, 38000, France; x Service de Médecine Intensive - Réanimation, APHM CHU Nord, Aix-Marseille Université, Marseille, France; y Département de Neurologie, Service de Médecine Intensive-Réanimation à Orientation Neurologique, Sorbonne Université, AP-HP, France; z Université Paris Cité, IAME, INSERM U1137, et APHP Nord, Médecine Intensive Réanimation, Hôpital Bichat - Claude Bernard, France; aa Sorbonne Université, Hôpital de la Pitié-Salpêtrière, 47-83, Boulevard de l'hôpital, Paris, 75013, France

**Keywords:** Meningitis, Mechanical ventilation, Intracranial pressure, Mortality

## Abstract

**Background:**

Community-acquired bacterial meningitis remains associated with high mortality and neurological disability in critically ill adults. Contemporary data on outcomes in mechanically ventilated patients and on the role of invasive intracranial pressure monitoring are limited. We aimed to describe 90-day outcomes, identify predictors of unfavorable neurological outcome, and evaluate the association between invasive intracranial pressure monitoring and prognosis in mechanically ventilated adults with community-acquired bacterial meningitis.

**Methods:**

Retrospective multicenter cohort study (2012–2023) across 26 French ICUs. The primary endpoint was unfavorable functional outcome at day 90 (modified Rankin Scale score 3–6), assessed using multivariable mixed-effects regression and propensity score–based overlap weighting.

**Findings:**

Among 704 included patients (median age, 66 years [IQR, 48–70 years]; 421 men [59.8%]), 354 (50.3%) had an unfavorable outcome at day 90, including 220 (31.3%) who died. Independent predictors of unfavorable outcome included older age, septic shock, acute kidney injury, coagulation disorders, lower motor score on the Glasgow Coma Scale, pupillary abnormalities, and abnormal brain imaging findings at presentation. Appropriate initial antimicrobial therapy, along with adjunctive dexamethasone, was associated with improved outcomes. Invasive intracranial pressure monitoring was performed in 84/704 patients (11.9%) and was not associated with improved functional outcome in the overall cohort (odds ratio, 0.97; 95% CI, 0.48–1.96) or in prespecified subgroups.

**Interpretation:**

In this large real-world cohort, 90-day mortality and disability remain high and are largely driven by initial neurological and systemic severity. Invasive intracranial pressure monitoring was not associated with improved outcomes, and no clear benefit was identified in this observational analysis.

**Funding:**

None.

**Research in context**

**Evidence before this study**

We searched PubMed and Embase from inception to May 2026, without language restriction, using the terms “bacterial meningitis”, “intracranial pressure monitoring”, “mechanical ventilation”, “intensive care”, and “outcome”. Reference lists of relevant systematic reviews were also screened. Studies were included if they reported mortality or functional outcomes in adult ICU patients with community-acquired bacterial meningitis; pediatric, nosocomial, and post-neurosurgical studies were excluded. Available evidence consisted predominantly of small single-center retrospective cohorts at moderate-to-high risk of bias, with no randomized controlled trial addressing ICP monitoring in this setting. The few observational studies included fewer than 80 monitored patients and relied on unadjusted comparisons. Evidence supporting ICP monitoring is largely extrapolated from traumatic brain injury, where protocolized strategies have shown inconsistent results. Reported 90-day mortality ranged from 25% to 45%, with unfavorable functional outcomes in 40–60%.

**Added value of this study**

Addressing this evidence gap, MAC-ICU is the largest multicenter cohort specifically designed to evaluate outcomes and the role of invasive intracranial pressure monitoring in mechanically ventilated adults with bacterial meningitis. Among 704 patients across 26 French ICUs, half experienced an unfavorable outcome at 90 days. Invasive ICP monitoring was not associated with improved neurological outcome after rigorous adjustment for confounding by indication, consistently across sensitivity analyses and prespecified subgroups.

**Implications of all the available evidence**

Early appropriate antimicrobial therapy and adjunctive dexamethasone remain central to prognosis. In current practice, invasive ICP monitoring was not associated with improved outcomes, and extrapolation from traumatic brain injury is not supported. Prospective randomized studies with protocolized ICP-guided strategies are needed to definitively address this question.

## Introduction

Over the past twenty years, significant advancements in vaccination strategies, molecular diagnosis, antimicrobial and adjuvant therapies, and critical care have greatly enhanced the management of community-acquired bacterial meningitis.^1^^,^^2^ Despite these improvements, bacterial meningitis remains a common and life-threatening disease worldwide, with over 2.5 million cases and approximately 300,000 deaths each year.^3^ Mortality rates are still high, ranging from 10% to 15%, and up to 25% of survivors experience long-term neurological or systemic complications.^4^

Patients with bacterial meningitis often need to be admitted to the intensive care unit (ICU), especially if they are in a coma, have refractory seizures, or experience systemic organ failure.^5^, ^6^, ^7^ In such cases, invasive mechanical ventilation, a clear indicator of disease severity, is consistently associated with poor outcomes.^6^^,^^8^^,^^9^ However, despite comprising patients with the highest neurological and systemic burden, this critically ill group has been studied in only a limited number of reports.

Neurological assessment in mechanically ventilated patients is particularly challenging. Sedation, often necessary to ensure adequate ventilation and control intracranial dynamics, limits the reliability of clinical examination and may delay recognition of secondary neurological deterioration. In this context, invasive intracranial pressure (ICP) monitoring is sometimes used to guide neuro-intensive care management when bedside evaluation and imaging provide limited information.^1^^,^^10^ While intracranial pressure (ICP) monitoring is recommended in current guidelines for other acute brain injuries,^11^ such as traumatic brain injury and hemorrhagic stroke, robust evidence supporting its use in comatose patients with bacterial meningitis remains scarce and controversial^1^ (Supplemental Table E1).

We therefore conducted the MAC-ICU study to identify predictors of unfavorable neurological outcome in patients with bacterial meningitis requiring invasive mechanical ventilation and to evaluate the impact of invasive ICP monitoring in this high-risk population.

## Methods

### Study design and participants

MAC-ICU is a retrospective, observational cohort study conducted across 26 French ICUs, encompassing both university and general hospitals, from January 1, 2012, to December 31, 2023. Centers’ characteristics are displayed in Supplemental Table E2. Patients were eligible for inclusion if they met the following prespecified criteria: (1) age ≥18 years; (2) community-acquired bacterial meningitis, defined by abnormal cerebrospinal fluid (CSF) findings, microbiological identification in CSF (positive Gram stain; positive CSF culture, positive CSF soluble antigen test or polymerase chain reaction) or isolation of a causative pathogen in blood cultures in a compatible clinical context and (3) receipt of invasive mechanical ventilation the day of ICU admission.

In each participating center, data were extracted from medical records by local investigators using a standardized case report form. Collected data included demographic characteristics, major comorbidities, symptoms before hospital admission, indications for endotracheal intubation, clinical presentation, physical examination findings at ICU admission, laboratory results, microbiological investigations, brain imaging findings during ICU stay, neurological monitoring and management, use of organ support therapies, follow-up, and outcomes. Data consistency and completeness were assessed before analysis, including evaluation of missing data, extreme values, and variable distributions (Supplemental Fig. E1).

### Study objectives

The primary objective was to report 90-day neurological status and to identify factors independently associated with unfavorable neurological outcome in a large, real-life cohort. The secondary objective was to assess the impact of invasive ICP monitoring use on neurological outcomes.

### Study outcome and definitions

The primary outcome was poor functional outcome 90 days after ICU admission, defined as a modified Rankin Scale (mRS) score of 3–6, indicating moderate-to-severe disability or death.^12^ The threshold of mRS ≥3 was selected to capture all clinically meaningful disability, consistent with prior trials in bacterial meningitis.^13^^,^^14^ Vital status at day 90 was determined through linkage with the French national death registry. For surviving patients, functional status was assessed from available medical records, including hospital discharge summaries and outpatient follow-up reports from relevant specialist consultations. For patients who died before day 90, the mRS was recorded as 6. Causes of death were classified into four categories by two investigators: brain death, according to the French legal neurological definition (combining comatose state, absent brainstem reflexes, and either two isoelectric EEGs or absent cerebral blood flow on imaging); refractory intracranial hypertension in the absence of brain death criteria, defined as clinical and/or radiological evidence of uncontrolled intracranial hypertension (pupillary abnormalities, brain herniation on imaging, absent brainstem reflexes), with or without invasive ICP measurement; withdrawal of life-sustaining therapy due to poor neurological recovery; and systemic causes, including multiorgan failure. Coma was defined as a Glasgow Coma Scale score (GCS) < 9. Septic shock was defined according to the Sepsis-3 criteria (sepsis-related organ dysfunction with vasopressor requirement and serum lactate>2 mmol/L),^15^ and acute kidney injury was defined according to the Kidney Disease: Improving Global Outcomes definition.^16^ Coagulation disorders were determined by the presence of thrombocytopenia (platelet count<100 × 10^9^/L) or disseminated intravascular coagulation according to the International Society on Thrombosis and Hemostasis criteria.^17^ The Sequential Organ Failure Assessment (SOFA) score was calculated using the worst values recorded within the first 24 h of admission. Appropriate initial antibiotic therapy was defined as the administration of at least one antibiotic active against the identified or suspected causative pathogen, initiated within the first 24 h after hospital admission. For culture-positive cases, appropriateness was defined by demonstrated in vitro susceptibility of the isolated pathogen. For cases diagnosed by PCR or antigen testing without available susceptibility data, appropriateness was defined as the use of an antibiotic with established activity against the identified species according to current guidelines.^18^^,^^19^ In microbiologically unconfirmed cases, appropriateness was assessed by the local investigator based on coverage of the most likely pathogens according to French guidelines for community-acquired bacterial meningitis. Empirical antibiotic regimens were described separately; vancomycin was not systematically recommended by French national guidelines during the study period, reflecting the low local prevalence of highly cephalosporin-resistant *Streptococcus pneumoniae*.^18^ Complications related to ICP monitor insertion were assessed, including procedure-related hemorrhage (defined as new intracranial bleeding on post-procedural neuroimaging attributed to the procedure) and device-related infection (positive CSF culture or clinical meningitis/ventriculitis without alternative source).

### Statistical analysis

Continuous variables were described as medians and interquartile ranges (IQR) and compared using the Wilcoxon’s rank sum test or the Kruskal–Wallis test; categorical variables were summarized by counts (percentage) and compared using the exact Fisher test.

Factors associated with poor functional outcome at day 90 were identified using multivariable analysis based on a mixed-effects regression model, with explanatory variables included as fixed effects and study center as a random effect to account for center-level variability. Candidate variables were selected a priori based on a review of the literature and the construction of a directed acyclic graph (Supplemental Fig. E2). Only variables available within 24 h after ICU admission were used. Brain imaging variables were restricted to abnormalities identified on the first neuroimaging performed within this timeframe, to ensure that only baseline predictors were considered. Findings from subsequent imaging were recorded for descriptive purposes only. The year of ICU admission and the use of an intracranial pressure monitor were pre-planned to be forced into the model. Log-linearity of continuous candidate variables was assessed using restricted cubic splines with 3 knots; no significant departure from linearity was identified and no transformations were applied. Collinearity between candidate variables was assessed using variance inflation factors on a standard logistic regression model.

### Effect of intracranial pressure monitoring on the modified Rankin scale

To estimate the causal effect of invasive ICP monitoring on the primary outcome, we used a propensity score-based inverse probability of treatment weighting approach with overlap weighting.^20^ The propensity score reflected each patient’s probability of receiving ICP monitoring conditional on clinically relevant confounders. Overlap weighting was applied to create a weighted pseudo-population with balanced baseline covariates between monitored and non-monitored patients, thereby reducing confounding by indication. Variables included in the propensity score model were age, Charlson comorbidity index, SOFA score, Glasgow Coma Scale before intubation, presence of a coagulation disorder, pupillary abnormalities, abnormal brain computed tomography findings, and the primary indication for endotracheal intubation. The association between ICP monitoring and outcome was assessed using weighted logistic regression models with robust standard errors. Center was included as a random effect to account for between-center variability. To avoid immortal time bias and heterogeneity, only intraparenchymal devices inserted within a 24-h window after ICU admission were considered. Covariate balance before and after weighting was assessed using standardized mean differences. Analyses were conducted in the overall weighted population and in prespecified subgroups, including patients with a Glasgow Coma Scale score<9, those intubated for coma (whatever the reason), patients with pupillary abnormalities, and patients with cerebral edema on brain imaging.

To account for missing data, multiple imputation by chained equations (MICE) was performed, generating 30 complete datasets using the *MICE* package. Analyses were conducted within each imputed dataset, and results were combined using Rubin’s rules. The measures of associations are presented with Odds ratios (OR) and 95% confidence intervals. All tests were two-sided, and p-values lower than 5% were considered to indicate significant associations. No formal correction for multiple testing was applied. Analyses were performed using R statistical platform, version 4.0.4 (https://cran.r-project.org/).

### Ethics approval

The study was approved by the Ethics Committee of the *Société de Réanimation de Langue Française* on September, 14, 2023 (IRB00014135, project n° 23–052). The need for informed consent was waived at every site for this retrospective study.

### Role of the funding source

The funder of the study had no role in study design, data collection, data analysis, data interpretation, or writing of the report.

## Results

### Patient characteristics

Among the 1871 patients admitted to the ICU for community-acquired bacterial meningitis, 704 patients (37.6%; 95% confidence interval [CI], 35.4–39.9%) required invasive mechanical ventilation and were included in the final analysis (Supplemental Figs. E3 and E4). Table 1 reports the baseline patient characteristics. The median age was 66 years [IQR 48–70], and 418 (59.8%) patients were male. One hundred and eighty-one patients (25.7%) were immunocompromised, and 113 (17.7%) had osteomeningeal defects. The median time from hospital admission to ICU transfer was 0 days [0–1]. All patients required invasive mechanical ventilation on the day of admission. The main reason for intubation was coma (n = 349, 40.5%) followed by agitation (n = 154, 21.8%) (Supplemental Fig. E5). A septic shock was present at admission in 182 (25.9%) patients, and 239 (36%) had coagulation disorders.

**Table 1:** Main baseline characteristics according to 90-day functional outcome in severe community-acquired bacterial meningitis.

	Missing Data N (%)	Overall N = 704 N (%) or Median [IQR]	Modified Rankin score <3 N = 350 N (%) or Median [IQR]	Modified Rankin score ≥3 N = 354 N (%) or Median [IQR]	p-value
Age- years	–	61 [48–70]	58 [43–68]	64 [54–72]	<0.001
Male sex	–	418 (59.8)	206 (59.4)	212 (60.2)	0.88
Charlson score	1 (0.1)	2 [1–4]	2 [0–3]	3 [1–5]	<0.001
Diabetes	1 (0.1)	141 (20.1)	58 (16.6)	83 (23.4)	0.030
Cardiovascular diseases	–	90 (12.8)	37 (10.6)	53 (15.0)	0.10
Chronic lung diseases	–	56 (8.0)	28 (8.0)	28 (7.9)	1.00
Chronic kidney diseases	–	45 (6.4)	14 (4.0)	31 (8.8)	0.015
Cirrhosis	–	34 (4.8)	4 (1.1)	30 (8.5)	<0.001
Alcohol abuse	2 (0.3)	163 (23.2)	65 (18.6)	98 (27.8)	0.005
IV drug use	–	33 (4.7)	18 (5.2)	15 (4.2)	0.68
Immunosuppression^a^	–				
Solid cancer		56 (8.1)	22 (6.3)	34 (9.6)	0.13
Hematological malignancies		49 (7.1)	26 (7.4)	23 (6.5)	0.74
Drug-related		38 (5.4)	18 (5.2)	20 (5.6)	0.90
HIV infection		22 (3.1)	11 (3.1)	11 (3.1)	1.00
Splenectomy		16 (2.3)	7 (2.2)	9 (2.5)	0.82
Osteomeningeal defect	–	113 (17.7)	77 (24.4)	36 (11.1)	<0.001
ICU admission					
Time hospital to ICU admission- days	2 (0.3)	0 [0–1]	0 [0-0]	0 [0–1]	0.006
Clinical manifestation					
Body Temperature- °c	33 (4.7)	38.5 [37.7–39.2]	38.6 [37.9–39.2]	38.5 [37.4–39.1]	0.028
Coma	17 (2.4)	441 (64.2)	219 (64.0)	222 (64.3)	0.99
Agitation and/or confusion	17 (2.4)	378 (53.7)	211 (60.3)	167 (47.2)	0.001
Seizures	32 (4.5)	149 (21.2)	77 (23.1)	72 (21.2)	0.62
Status epilepticus	37 (5.3)	38 (5.7)	15 (4.5)	23 (6.8)	0.26
Hemiparesis/hemiplegia	40 (5.7)	87 (13.1)	31 (9.4)	56 (16.8)	0.007
Cranial nerves involvement	37 (5.3)	62 (9.3)	28 (8.4)	34 (10.1)	0.53
Pupillary abnormalities	37 (5.3)	121 (18.1)	46 (13.9)	75 (22.3)	0.007
Unilateral mydriasis		39 (43.8)	9 (25.0)	22 (41.5)	
Bilateral mydriasis		31 (34.8)	16 (44.4)	23 (43.4)	
Miosis		19 (21.3)	11 (30.6)	8 (15.1)	
Glasgow coma scale	25 (3.6)	8 [5–10]	8 [6–10]	7 [3–10]	0.006
Motor component	27 (3.8)	4 [3–5]	5 [4–5]	4 [1–5]	0.001
Septic shock	–	182 (25.9)	51 (14.6)	131 (37.0)	<0.001
Acute respiratory failure	–	120 (17.0)	50 (14.3)	70 (19.8)	0.07
Coagulation disorders	40 (5.7)	239 (36.0)	86 (25.9)	153 (46.1)	<0.001
Acute kidney injury	12 (1.7)	216 (31.2)	66 (19.0)	150 (43.5)	<0.001
Mean arterial pressure- mmHg	77 (10.9)	83 [68–99]	87 [70–100]	81 [65–98]	0.018
Vasopressor use- day of admission	10 (1.4)	304 (43.8)	124 (35.8)	180 (51.7)	<0.001
Laboratory values- day of admission (worst values)					
Arterial blood gas					
PaO_2_ - mmHg	42 (6.0)	96 [75–131]	98 [78–136]	92 [73–125]	0.017
FiO_2_- %	98 (13.9)	40 [30–60]	36 [31–41]	35 [28–42]	0.21
PaCO_2_- mmHg	49 (7.0)	36 [30–41]	40 [30–50]	40 [30–60]	<0.001
Lactates- mmol/l	73 (10.4)	2.3 [1.5–3.8]	2.0 [1.3-3.1]	2.6 [1.8–4.5]	<0.001
Plasma sodium- mmol/l	17 (1.4)	137 [133–140]	137 [134–140]	136 [133–140]	0.30
Creatinine- μmol/l	12 (1.7)	82 [63–139]	75 [59–100]	98 [69–179]	<0.001
Platelet count- G/l	28 (4.0)	165 [89–224]	177 [124–233]	144 [69–213]	<0.001
Prothrombin time- %	42 (6.0)	67 [55–79]	71 [60–84]	62 [50–74]	<0.001
Blood glucose- mmol/l	72 (10.2)	8.7 [7.1–10.8]	8.8 [7.4–10.9]	8.5 [7.0–10.8]	0.18
SOFA score at admission	48 (6.8)	8 [5–11]	7 [5–9]	9 [6–13]	<0.001
Microbiology					
Lumbar puncture^b^	–	685 (98.1)	345 (98.6)	340 (97.4)	0.42
CSF examination results					
Leukocytes count-/μl	49 (7.0)	1150 [240–4550]	1971 [459–5650]	800 [158–3410]	<0.001
Protein- g/l	78 (11.1)	5.0 [2.8–7.8]	4.6 [2.9–7.2]	5.5 [2.5–8.4]	0.08
Glucose- mmol/l	106 (15.1)	0.3 [0.1–2.4]	0.4 [0.1–2.7]	0.3 [0.1–2.1]	0.99
Lactates- mmol/l	474 (67.3)	13.9 [10.0–17.9]	13.3 [9.3–16.7]	15 [11.2–19.3]	0.013
Positive direct examination	13 (1.8)	498 (72)	248 (71.5)	250 (72.7)	0.79
Positive culture	19 (2.7)	476 (69.5)	236 (68.2)	240 (70.8)	0.51
Positive soluble antigen	53 (7.5)	209 (32.1)	97 (30.8)	66 (21.5)	0.011
Positive PCR	82 (11.6)	163 (26.2)	107 (32.7)	102 (31.5)	0.80
Positive blood culture	5 (0.7)	426 (60.9)	193 (55.3)	233 (66.6)	0.003
First antimicrobial therapy on hospital admission					
Third-generation cephalosporin	–	659 (92.3)	345 (98.6)	314 (89.2)	<0.001
Amoxicillin	–	246 (34.9)	131 (39.8)	115 (34.6)	0.19
Acyclovir	–	221 (31.4)	114 (35.1)	107 (32.3)	0.51
Adequate initial antibiotic therapy	5 (0.7)	624 (89.3)	331 (95.4)	293 (83.2)	<0.001
Adjuvant Dexamethasone	–	507 (72)	283 (80.9)	224 (63.3)	<0.001

Data are presented as medians and interquartile ranges [IQR], unless otherwise indicated.

p-values were calculated for comparisons between patients who had a Modified Rankin score ≥3 at day-90 and those who did not.

CSF, cerebrospinal fluid; FiO_2_, Fraction of inspired oxygen; HIV: human immunodeficiency virus; IV, intravenous; ICU, intensive care unit; PaO_2_, partial pressure of oxygen; PaCO_2_, partial pressure of carbon dioxide; PCR, polymerase chain reaction; SOFA, Sequential Organ Failure Assessment.

a Percentages may not exactly total 100% because of non-exclusive categories.

b Not done reason: severe coagulation disorders (n = 7), elevated intracranial pressure (n = 6).

Regarding neurological presentation, the median GCS score was 8 [5–10] before intubation. Hemiplegia or hemiparesis was present in 87 patients (13.1%), and 149 patients (21.2%) experienced convulsive seizures.

**Fig. 1:**
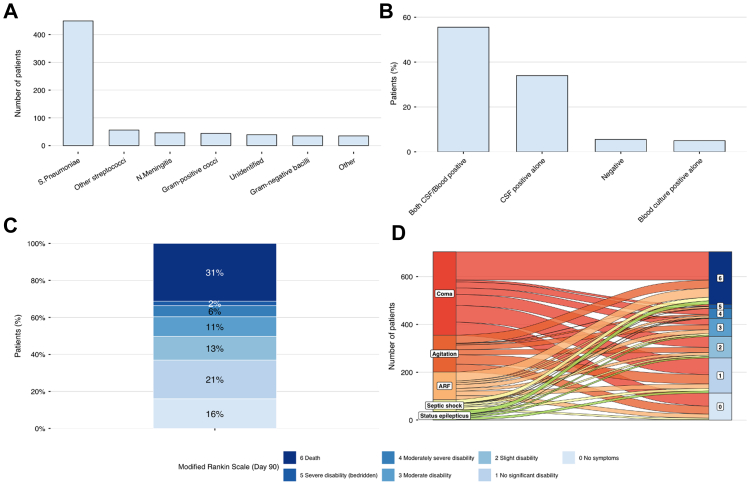
**Microbiology, clinical presentation, and 90-day outcomes.** Distribution of causative pathogens (panel A), Microbiological positivity according to sampling site (panel B), Modified Rankin Scale distribution at Day 90 (panel C), and Sankey diagram showing trajectories from main reasons for intubation indication to Day-90 functional outcome (panel D). The flow width is proportional to the number of patients. ARF: acute respiratory failure; CSF: cerebrospinal fluid.

The most commonly identified causative pathogen was *S. pneumoniae* (n = 449, 63%; Fig. 1A). Microbiological investigations are summarized in Fig. 1B and Supplemental Fig. E6. Lumbar puncture was performed in 685 patients (98.1%). Reasons for lumbar puncture avoidance included severe coagulation disorders (n = 7) and suspected or confirmed elevated intracranial pressure (n = 6). At ICU admission, third-generation cephalosporins were administered in 659 patients (92.3%), amoxicillin in 246 (34.9%), and acyclovir in 221 (31.4%) (Table 1 and Supplemental Fig. E7). Initial antibiotic therapy was considered adequate in 624 patients (89.3%), and adjunctive dexamethasone was used in 507 patients (72.0%).

During the ICU stay, the most frequent complications were ventriculitis (n = 100, 14.2%), followed by vasculitis (n = 82, 11.7%), brain abscess (n = 70, 10.0%), and empyema (n = 34, 4.9%). Associated endocarditis was observed in 52 patients (7.4%), including 22 cases (3.1%) attributable to *S. pneumoniae* (Austrian syndrome).

Table 2 summarizes neurological monitoring and ICU management throughout ICU stay. Neuroimaging was performed in 685 (98.1%) patients. The most frequently observed brain imaging abnormalities were cerebral ischemia (n = 187, 26.6%) and brain edema (n = 135, 19.3%). During the ICU stay, ICP monitoring was performed in 84 patients (11.9%), and CSF drainage was used in 66 patients (9.4%). The median time from ICU admission to ICP monitoring and CSF drainage was 0.0 [0.0–1.0] days and 1.0 [0.0–4.0] days, respectively.

**Table 2:** ICU management and outcomes according to 90-day functional outcome in severe community-acquired bacterial meningitis.

	Missing Data N (%)	Overall N = 704 N (%) or Median [IQR]	Modified Rankin score <3 N = 350 N (%) or Median [IQR]	Modified Rankin score ≥3 N = 354 N (%) or Median [IQR]	p-value
Neuro-monitoring					
Brain imaging	–	679 (96.6)	341 (97.4)	338 (95.8)	0.31
CT scan	–	656 (93.2)	327 (93.4)	329 (92.9)	0.91
MRI	–	317 (45.0)	149 (42.6)	168 (47.5)	0.22
Abnormal brain imaging^a^		396 (56.2)	155 (44.3)	241 (68.1)	<0.001
Cerebral edema	5 (0.7)	135 (19.3)	44 (12.7)	91 (25.8)	<0.001
Brain herniation	5 (0.7)	36 (5.2)	7 (2.0)	29 (8.2)	<0.001
Hydrocephalus	5 (0.7)	81 (11.6)	23 (6.6)	58 (16.4)	<0.001
Venous Thrombosis	5 (0.7)	63 (9.0)	29 (8.4)	34 (9.6)	0.66
Ischemia	5 (0.7)	187 (26.6)	54 (15.4)	133 (37.6)	<0.001
Localized		130 (18.6)	40 (11.6)	90 (25.5)	
Diffuse		57 (8.2)	14 (4.0)	43 (12.2)	
Hemorrhage	5 (0.7)	75 (10.7)	19 (5.4)	56 (15.8)	<0.001
Localized		60 (8.6)	16 (4.6)	44 (12.5)	
Diffuse		15 (2.1)	3 (0.9)	12 (3.4)	
Meningitis complications^a^					
Empyema	6 (0.9)	34 (4.9)	12 (3.5)	22 (6.2)	0.13
Abscess	6 (0.9)	70 (10.0)	26 (7.5)	44 (12.5)	0.039
Ventriculitis^c^	6 (0.9)	100 (14.2)	44 (12.6)	56 (15.9)	0.25
Vasculitis	4 (0.6)	82 (11.7)	24 (6.9)	58 (16.4)	<0.001
EEG performed	10 (1.4)	418 (60)	180 (52.5)	238 (67.8)	<0.001
Transcranial doppler		227 (32.3)	110 (31.5)	117 (33.1)	0.70
Abnormal Transcranial doppler^b^, ^c^, ^d^, ^e^		157 (68.9)	73 (65.8)	84 (71.8)	0.40
Pulsatility index- right		1.5 [1.1-1.9]	1.5 [1.1-1.9]	1.5 [1.1–2.0]	0.63
Pulsatility index- left		1.4 [1.1-1.9]	1.4 [1.1-1.9]	1.4 [1.1–2.0]	0.50
Intracranial pressure monitoring	–	84 (11.9)	41 (11.7)	43 (12.1)	0.95
Time ICU admission-ICP monitoring- days	–	0.0 [0.0–1.0]	0.0 [0.0–1.0]	1.0 [0.0–1.0]	0.16
Operator	–				
Neurosurgeon		47 (58.8)	24 (58.5)	23 (59.0)	1.00
Coagulation disorders at monitor insertion	–	13 (14.9)	4 (9.3)	9 (20.5)	0.25
Transfusion for monitor insertion		10 (11.5)	3 (7.0)	7 (15.9)	0.33
ICP value (on day 1)- mmHg	8 (0.1)	25 [13–35]	23 [13–27]	30 [13–46]	0.030
ICP monitoring duration- days	12 (14.3)	4.0 [2.0–8.0]	4.0 [3.0–7.0]	3.0 [2.0–9.0]	0.20
ICP monitor bleeding	–	3 (3.6)	2 (4.9)	1 (2.3)	0.97
ICP monitor infection	–	1 (1.2)	0 (0.0)	1 (2.3)	1.00
CSF drainage	–	66 (9.4)	23 (6.6)	43 (12.1)	0.016
External ventricular drainage		65 (9.6)	22 (6.5)	43 (12.6)	0.010
External lumbar drainage		2 (0.3)	1 (0.3)	1 (0.3)	1.00
Time ICU admission- CSF drainage- days	2 (0.3)	1.0 [0.0–4.0]	0.0 [0.0–1.0]	1.0 [1.0–6.8]	0.004
Operator	–				
Neurosurgeon		64 (98.5)	23 (100.0)	41 (97.6)	1.00
Coagulation disorders at insertion		11 (17.5)	3 (13.0)	8 (20.0)	0.72
Transfusion for insertion	7 (10.6)	5 (8.3)	2 (8.7)	3 (8.1)	1.00
CSF drainage duration- days	4 (0.1)	11 [7–20]	8 [7–11.5]	14 [8–24]	0.028
Intraventricular antibiotics	–	6 (9.1)	2 (8.7)	4 (9.3)	1.00
CSF drainage bleeding	–	4 (6.1)	2 (8.7)	2 (4.7)	0.91
CSF drainage infection	–	6 (9.1)	3 (13.0)	3 (7.0)	0.71
ICU stay management					
Invasive mechanical ventilation	–	704 (100.0)	350 (100.0)	354 (100.0)	–
Time ICU admission to iMV-days	–	0 [0-0]	0 [0-0]	0 [0-0]	0.07
iMV duration- days	14 (2.0)	6 [3–12]	4 [3–8]	8 [3–17]	<0.001
Vasopressor use	10 (1.4)	377 (54)	148 (42.4)	229 (64.7)	<0.001
Inotropes use	–	45 (6)	15 (4.3)	30 (8.5)	0.034
Renal replacement therapy use	6 (0.9)	108 (16)	22 (6.3)	86 (24.6)	<0.001
Any surgical intervention^d^	–	82 (11.6)	37 (10.6)	45 (12.7)	0.41
Outcomes					
Day-90 Modified Rankin score	–	3 [1–6]	1 [0–2]	6 [4–6]	<0.001
Cause of death at Day-90	–				
Multiorgan failure/sepsis		77 (35.0)	–	77 (35.0)	
Poor neurological status recovery		64 (29.1)	–	64 (29.1)	
Brain death		36 (16.4)	–	36 (16.4)	
Refractory intracranial hypertension		35 (15.9)	–	35 (15.9)	
Others causes^e^		8 (3.6)	–	8 (3.6)	
ICU mortality	–	194 (27.6)	0 (0.0)	194 (54.8)	<0.001
ICU length of stay- days	5 (0.7)	8 [5–16]	7 [5–12]	11 [4–23]	<0.001
Hospital mortality	–	219 (33.6)	0 (0.0)	219 (65.4)	<0.001
Hospital length of stay- days	82 (11.6)	19 [11–38]	20 [13–37]	16 [6–38]	<0.001

Data are presented as medians and interquartile ranges [IQR], unless otherwise indicated.

p-values were calculated for comparisons between patients who had a Modified Rankin score ≥3 at day-90 and those who did not.

CSF, cerebrospinal fluid; CT-scan, computed tomography scanner; EEG, Electroencephalography; ICP, intracranial pressure monitoring; ICU, intensive care unit; iMV, invasive mechanical ventilation; MRI, magnetic resonance imaging.

a Percentages may not exactly total 100% because of non-exclusive categories.

b Defined as a pulsatility index >1.3 and/or abnormal mean flow velocities consistent with intracranial hypertension or cerebral vasospasm, and/or marked asymmetry between cerebral arteries.

c Ventriculitis was diagnosed predominantly in patients who underwent MRI, reflecting an imaging-dependent detection bias. Its prevalence should be interpreted with caution given the heterogeneity in MRI use across centers.

d Including ENT interventions (n = 23, 28.0%), valvular replacement (n = 17, 20.7%), abscess/joint washout (n = 12, 14.6%), CNS interventions (n = 11, 13.4%), surgery for digestive ischemia (n = 8, 9.8%), empyema drainage (n = 5, 6.1%), ventriculoperitoneal shunt placement (n = 4, 4.9%), and pericardiocentesis (n = 2, 2.4%).

e Including 2 severe nosocomial infections, 2 withdrawal of care because of advanced malignancy diagnosis, one fatal pulmonary embolism, and one suicide attempt. Two causes were unknown.

### Factors associated with unfavorable outcome at day 90

At day 90, 354 patients (50.3% [46.5–54.0%]) had an unfavorable outcome (Fig. 1C), including 220 deaths (31.3%). The leading causes of death were multiorgan failure (n = 77, 35.0%) and withdrawal of life-sustaining therapy due to poor neurological recovery (n = 64, 29.1%). Fig. 1D illustrates the association between the primary indication for endotracheal intubation and functional outcome. As shown, a comatose state as the main indication for intubation was associated with the poorest outcome.

**Fig. 2:**
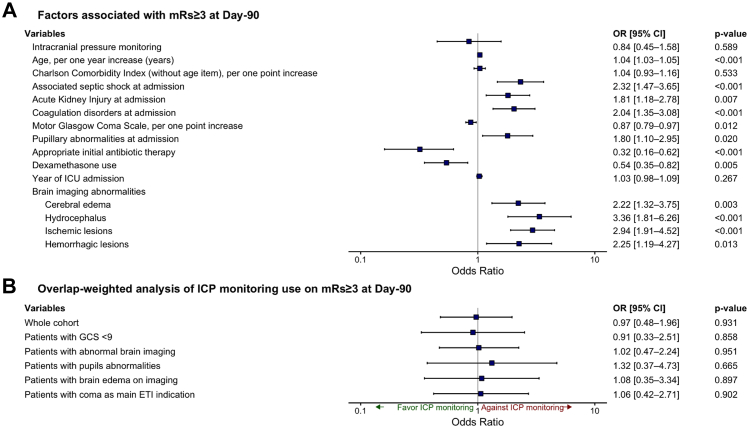
**Forest plots showing factors associated with a modified Rankin Scale (mRS) score ≥3 at day 90 (panel A) and the effect of invasive intracranial pressure monitoring on mRS ≥3 in the overall population and predefined subgroups (panel B).** All estimates are mutually adjusted for all covariates displayed. Center was included as a random effect. The between-center variance in the primary multivariable model was 0.119 (SD: 0.345), corresponding to an intraclass correlation coefficient of 0.035. GCS: Glasgow coma scale; ETI: endotracheal intubation.

Factors associated with day-90 unfavorable outcome in univariable analysis are described in Tables 1 and 2. By multivariable analysis, several factors were independently associated with poor functional outcome at day 90 (Fig. 2A). Increasing age (odds ratio per one-year increase, 1.04 [1.03–1.05]), septic shock at ICU admission (2.32 [1.47–3.65]), acute kidney injury (1.81 [1.18–2.78]), and coagulation disorders at admission (2.04 [1.35–3.08]) were associated with worse outcomes. Markers of neurological severity were also strongly associated with outcome, including lower Glasgow Coma Scale motor score at admission (OR per one-point increase, 0.87 [0.79–0.97]), pupillary abnormalities at admission (1.80 [1.10–2.95]) and brain imaging abnormalities: cerebral edema (2.22 [1.32–3.75]), hydrocephalus (3.36 [1.81–6.26]), ischemic lesions (2.94 [1.91–4.52]), and hemorrhagic lesions (2.25 [1.19–4.27]). Conversely, appropriate initial antibiotic therapy (0.32 [0.16–0.62]) and dexamethasone use (0.54 [0.35–0.82]) were associated with a lower risk of poor functional outcome, whereas intracranial pressure monitoring was not (0.84 [0.45–1.58]).

No association was found between the identified pathogen and outcome (data not shown).

### Association between intracranial pressure monitoring and modified Rankin scale

Overall, ICP monitoring was used in 84 (11.9% [9.6–14.6%]) patients. At the time of catheter insertion, 52 patients (63.4%) had an ICP ≥20 mmHg. Baseline characteristics of patients managed with and without invasive ICP monitoring are presented in Table 3. As shown, patients monitored were younger, had fewer comorbidities, more frequent pupillary abnormalities, abnormal brain imaging, and a lower prevalence of coagulation disorders. In addition, the use of deep sedation, hyperosmolar agents, external cerebrospinal fluid drainage, corticosteroids, and selected nonpharmacological therapies was more frequent during the ICU stay among patients who underwent ICP monitoring (Table 3). No major complications at insertion or during ICU stay were noticed (Table 2). No significant temporal trend in ICP monitoring use was found over the study period (chi-squared test for trend p = 0.98, Supplemental Fig. E8).

**Table 3:** Baseline characteristics, ICU management, and outcomes according to intracranial pressure monitoring.

	No Intracranial pressure monitoring N = 620 N (%) or Median [IQR]	Intracranial pressure monitoring N = 84 N (%) or Median [IQR]	p-value
Age- years	62 [49–70]	54 [40–67]	<0.001
Male sex	373 (60.6)	45 (54.2)	0.32
Charlson score	2 [1–4]	1 [0–3]	<0.001
Time for hospital to ICU admission- days	0 [0–1]	0 [0-0]	0.07
Clinical manifestation			
Body temperature- °c	38.5 [37.6–39.1]	38.9 [38.1–39.4]	0.024
Coma	390 (64.6)	51 (61.4)	0.66
Confusion	306 (50.8)	36 (43.4)	0.25
Agitation	183 (30.7)	29 (35.4)	0.47
Agitation and/or Confusion	338 (54.5)	40 (47.6)	0.20
Seizures	133 (22.5)	16 (19.5)	0.63
Status epilepticus	36 (6.2)	2 (2.4)	0.27
Hemiparesis/hemiplegia	79 (13.6)	8 (9.8)	0.4
Cranial nerves involvement	15 (2.6)	1 (1.2)	0.72
Pupillary abnormalities	49 (8.4)	13 (15.9)	0.048
Glasgow coma scale	8 [5–10]	7 [5–9]	0.14
Motor component	4 [2–5]	4 [3–5]	0.42
Septic shock	160 (25.8)	22 (26.2)	1.00
Coagulation disorders	225 (38.3)	14 (18.4)	0.001
Acute kidney injury	201 (33.0)	15 (18.1)	0.009
Mean arterial pressure- mmHg	83 [67–98]	87 [71–101]	0.20
Vasopressor use- day of admission	258 (42.3)	46 (54.8)	0.041
Laboratory values- day of admission (worst values)			
Arterial blood gas			
PaO_2_ - mmHg	95 [74–131]	102 [81–134]	0.08
FiO_2_- %	35 [30–40]	40 [30–50]	0.25
PaCO_2_- mmHg	40 [30–60]	40 [30–50]	0.87
Lactates- mmol/l	2.3 [1.5–3.7]	2.4 [1.6–3.8]	0.58
Plasma sodium- mmol/l	136 [133–140]	137 [134–140]	0.36
Platelet count- G/l	159 [84–220]	191 [147–258]	<0.001
Prothrombin time- %	66 [55–79]	70 [60–81]	0.08
Blood glucose- mmol/l	8.7 [7.0–10.9]	8.4 [7.8–10.5]	0.74
SOFA score at admission	7 [5–11]	8 [5–10]	0.87
Microbiology			
Lumbar puncture^b^	602 (97.7)	83 (100.0)	0.33
Positive blood culture	387 (62.7)	39 (47.6)	0.012
Pathogen identified			0.10
*Streptococcus Pneumoniae*	388 (62.6)	61 (72.6)	
Adequate initial antibiotic therapy	551 (89.6)	73 (86.9)	0.58
Adjuvant dexamethasone	440 (71.0)	67 (79.8)	0.12
Neuro-monitoring			
Brain imaging	595 (96.1)	84 (100.0)	0.13
Abnormal brain imaging	324 (52.3)	72 (85.7)	<0.001
EEG performed	357 (58.6)	56 (66.7)	0.20
Transcranial doppler	154 (24.9)	73 (86.9)	<0.001
Abnormal transcranial doppler^a^	93 (60.0)	64 (87.7)	<0.001
Pulsatility index- right	1.4 [1.0–1.8]	1.6 [1.4–2.0]	0.001
Pulsatility index- left	1.2 [1.0–1.8]	1.6 [1.3–2.0]	0.004
Neurological care management			
Deep sedation	205 (33.1)	72 (85.7)	<0.001
High doses corticosteroids	48 (9.0)	19 (22.9)	<0.001
Elevated ICP Management			
Hyperosmolar therapy	33 (6.2)	43 (51.8)	<0.001
External Ventricular/lumbar drain	31 (5.0)	35 (41.7)	<0.001
Subtractive Lumbar puncture	13 (2.3)	1 (1.2)	0.81
Mild hypocapnia	4 (0.8)	5 (6.0)	0.001
Neuromuscular paralysis	32 (6.0)	33 (39.8)	<0.001
Hypothermia	15 (2.8)	21 (25.3)	<0.001
Pentobarbital/Thiopentone coma	15 (3.0)	15 (18.3)	<0.001
Decompressive Craniectomy	2 (0.4)	5 (6.0)	<0.001
ICU stay management			
Invasive mechanical ventilation	620 (100.0)	84 (100.0)	–
iMV duration- days	5 [3–11]	9 [5–16]	<0.001
Vasopressor use	318 (51.4)	59 (70.2)	0.002
Inotropes use	34 (5.5)	11 (13.1)	0.015
Renal replacement therapy use	101 (16.4)	7 (8.6)	0.10
Outcomes			
Day-90 Modified Rankin score	3 [1–6]	3 [1–6]	0.60
ICU mortality	170 (27.4)	24 (28.6)	0.93
ICU length of stay- days	8 [4–16]	13 [7–22]	0.001
Hospital mortality	193 (33.8)	26 (32.1)	0.86
Hospital length of stay- days	18 [11–37]	21 [10–43]	0.55

Data are presented as medians and interquartile ranges [IQR], unless otherwise indicated. p-values were calculated for comparisons between patients who have received invasive intracranial monitoring and those who did not.

CSF, cerebrospinal fluid; CT-scan, computed tomography scanner; EEG, Electroencephalography; FiO_2_, Fraction of inspired oxygen; ICP, intracranial pressure monitoring; ICU, intensive care unit; iMV, invasive mechanical ventilation; MRI, magnetic resonance imaging; PaO_2_, partial pressure of oxygen; PaCO_2_, partial pressure of carbon dioxide.

a Defined as a pulsatility index >1.3 and/or abnormal mean flow velocities consistent with intracranial hypertension or cerebral vasospasm, and/or marked asymmetry between cerebral arteries.

b Not done reason : severe coagulation disorder (N = 7), elevated intracranial pressure (N = 6).

To address confounding by indication related to ICP monitoring, we estimated its effect on functional outcome at day 90 using a propensity score-based overlap weighting approach. Diagnostics of the propensity score model and weighting procedure are reported in the Supplemental Appendix (Supplemental Figs. E9 and E10).

In the overall weighted population, ICP monitoring was not associated with a reduced risk of poor functional outcome at day 90 (OR: 0.97 [0.48–1.96]; p = 0.93). These findings were consistent across all prespecified subgroups examined to assess potential heterogeneity of effect according to baseline neurological severity (Fig. 2B). In sensitivity analyses restricted to centers performing ICP monitoring, as well as to patients who received either intraparenchymal devices or ventricular catheters inserted within 24 h after admission (n = 103), results were consistent with the primary analysis (OR 0.97 [0.47–2.02], p = 0.95 and OR 1.02 [0.53–1.96], p = 0.96, respectively). Finally, the impact of 24/7 on-site neurosurgical availability (21 centers) was assessed using complementary approaches. Inclusion of this variable in the multivariable model showed no association with functional outcome (OR for 24/7 on-site neurosurgical availability: 1.21 [0.75–1.94]; p = 0.44). Consistent results were observed in propensity score analyses restricted to centers with on-site neurosurgery (OR for ICP monitoring: 1.00 [0.47–2.12]; p = 0.99) and to those without (OR for ICP monitoring: 0.96 [0.46–2.00]; p = 0.92). Overall, on-site neurosurgical availability did not materially influence the study findings.

### Post-hoc analyses

Post hoc analyses using alternative outcome definitions (mRS ≥4 and ordinal mRS) yielded results consistent with the primary analysis, with no association between ICP monitoring and outcome across all models (multivariable OR = 0.92 [0.49–1.72]; overlap weighting OR = 1.08 [0.53–2.21]; ordinal model OR = 0.82 [0.42–1.59]). Results are detailed in the Supplemental Appendix.

To assess the potential impact of unmeasured confounding, E-values were calculated. The E-value for the confidence interval limit closest to the null was 1.75 for the multivariable estimate and 1.97 for the overlap weighting estimate, indicating that an unmeasured confounder would need to be associated with both ICP monitoring and functional outcome by a risk ratio of at least this magnitude to fully explain the observed associations.

## Discussion

In this large multicenter cohort of mechanically ventilated adults with community-acquired bacterial meningitis, half of patients experienced an unfavorable functional outcome at 90 days. Prognosis was primarily driven by the initial neurological and systemic burden, and invasive intracranial pressure monitoring was not associated with improved recovery. Conversely, early appropriate antimicrobial therapy and adjunctive dexamethasone were associated with better outcomes, underscoring the central role of disease-modifying interventions in this setting.

*S. pneumoniae* remained the predominant pathogen, accounting for nearly two-thirds of cases, consistent with recent Western cohorts, despite widespread vaccination.^3^^,^^7^, ^8^, ^9^ The high proportion of immunocompromised patients and those with osteomeningeal defects further highlights the vulnerability of this population.

Outcomes were primarily driven by initial neurological injury and systemic severity.^6^^,^^8^^,^^13^ Older age, septic shock, acute kidney injury, and coagulation disorders were strongly associated with poor functional outcome and accounted for a substantial proportion of deaths.^21^ Neurological severity at presentation was a major determinant of prognosis, with impaired motor response, pupillary abnormalities, and structural brain lesions on imaging consistently associated with unfavorable outcomes, in line with previous studies.^21^^,^^22^ These findings support the concept of severe bacterial meningitis as a diffuse inflammatory and cerebrovascular disease rather than a purely meningeal infection.^22^^,^^23^

The association between early, appropriate antimicrobial therapy and improved outcomes in our study is consistent with previous evidence showing that delays in antibiotic administration increase mortality and neurological sequelae in bacterial meningitis.^9^ Adjunctive dexamethasone, when administered early alongside adequate antimicrobial therapy, has also been shown to reduce unfavorable neurological outcomes, particularly in pneumococcal meningitis.^24^ Although dexamethasone was associated with worse outcomes in Listeria meningitis in an earlier cohort study,^25^ this finding has been subsequently challenged, with confounding by indication proposed as a likely explanation^26^; given the limited number of Listeria cases in our cohort, pathogen-specific analyses were not feasible. Recent population-based studies further confirm that adherence to early, evidence-based management strategies is strongly associated with improved survival and functional recovery.^27^ Together, these findings highlight that timely anti-infective and anti-inflammatory treatment remains central to prognosis, despite advances in neuro-intensive care.^1^^,^^2^

Despite the strong prognostic value of structural brain injury, the clinical benefit of ICP monitoring in severe bacterial meningitis remains controversial.^1^^,^^28^ In practice, its use is largely driven by the limited reliability of neurological examination under deep sedation and the poor correlation between brain imaging findings and actual intracranial pressure.^23^ However, available evidence mainly relies on small single-center cohorts, retrospective series, or extrapolation from other acute brain injuries, with heterogeneous indications and outcome measures.^10^^,^^29^ Recent neurocritical care studies suggest that ICP monitoring may facilitate the detection of intracranial hypertension and guide targeted interventions,^30^ but have failed to demonstrate a consistent impact on functional outcome or survival after adjustment for baseline severity.^28^^,^^29^ In this context, our study provides the largest multicenter evaluation of invasive ICP monitoring in mechanically ventilated adults with community-acquired bacterial meningitis. Despite frequent documentation of intracranial hypertension and extensive use of ICP-guided therapies, we found no association between ICP monitoring and improved neurological outcome at 90 days, either in the overall population or in prespecified subgroups.

These findings were consistent after adjustment for confounding by indication and in sensitivity analyses restricted to centers routinely using ICP monitoring. However, important caveats remain. Residual confounding is likely, as monitored patients differed at baseline and received more intensive neuro-critical care, reflecting treatment selection driven by perceived neurological severity that is only partially captured by available variables. Accordingly, our results should be interpreted as an absence of demonstrated association rather than evidence of no effect. In addition, ICP monitoring is not a therapeutic intervention, and its potential benefit depends on how the information is used to guide treatment. In the absence of standardized protocols, heterogeneity in clinical responses may have contributed to the findings. Whether protocolized ICP-guided strategies improve outcomes in bacterial meningitis remains an open question.

Taken together, our results suggest that while ICP monitoring may refine physiological assessment and inform neuro-intensive care management, it does not appear to modify long-term neurological prognosis in severe bacterial meningitis. This contrasts with interventions targeting the primary infectious and inflammatory processes and highlights the need for caution when extrapolating ICP-based strategies from other acute brain injuries.^31^ Similarly, targeted temperature management (used in 5.1% of patients in our cohort, primarily for elevated intracranial pressure) should be distinguished from the systematic therapeutic hypothermia shown to be harmful in the trial by Mourvillier et al.,^32^ and its use as an adjunct to ICP-directed therapy in this setting remains poorly evidenced.

Future studies should focus on identifying subgroups most likely to benefit from advanced neuromonitoring and on integrating physiological monitoring with targeted therapeutic strategies addressing the underlying infectious and inflammatory processes.

Strengths of our study include (1) the large, multicenter cohort encompassing 26 ICUs, providing the largest real-world evaluation to date of severe community-acquired bacterial meningitis requiring mechanical ventilation; (2) the focus on a well-defined, clinically homogeneous population representing the most severe forms of the disease; and (3) the consistency of findings across multiple sensitivity analyses, including propensity score-based overlap weighting and restriction to centers routinely using ICP monitoring.

This study has several limitations. First, its observational design precludes definitive causal inference, and residual confounding related to unmeasured clinical decision-making cannot be excluded, despite the use of robust causal inference methods. Although ICP monitoring was restricted to devices inserted within 24 h of ICU admission to limit immortal time bias, a formal landmark analysis was not performed. However, the low rate of very early death in the non-monitored group (10.3% within 24 h) and the early timing of device insertion suggest that any such bias is unlikely to have materially influenced the results. Second, by restricting the analysis to mechanically ventilated patients, generalizability to less severe meningitis is limited, although this choice reflects the population in whom neurological assessment is most challenging. Third, detailed longitudinal intracranial pressure data and standardized ICP-guided management protocols were unavailable, preventing assessment of whether specific pressure patterns or interventions might benefit selected patients. Fourth, functional outcome was assessed at 90 days; neurological recovery may continue beyond this time point and thus may not be fully captured.^4^^,^^33^ Withdrawal of life-sustaining therapy accounted for a substantial proportion of deaths, raising the possibility of bias related to prognostic pessimism. However, the distribution of causes of death according to monitoring status did not support a self-fulfilling prophecy, as withdrawal for poor neurological prognosis was less frequent among monitored patients (data not shown). Finally, the relatively low proportion of patients receiving invasive ICP monitoring may introduce selection bias, although this likely reflects current practice and the limited adoption of this strategy.

In conclusion, in this large contemporary cohort of mechanically ventilated adults with community-acquired bacterial meningitis, outcomes were primarily driven by initial neurological and systemic severity, whereas invasive intracranial pressure monitoring was not associated with improved functional outcomes after adjustment for measured confounders. These findings highlight the central role of early disease-modifying interventions and suggest that the potential benefit of invasive neuromonitoring remains uncertain, warranting further prospective evaluation.

## Contributors

GD, CP, RS designed and performed research; GD, CP analyzed the data; GD, CP wrote the manuscript; CP, NT, EC, LA, SJ, MLP, JRL, HH, RT, PL, DB, PB, JM, JJ, GN, FA, JB, MB, MD, AC, BB, BC, SH, LLG, EF, NR, RS, and GD collected the data.

## Data sharing statement

The datasets analyzed during the current study are available from the corresponding author upon reasonable request (deidentified participant data, data dictionary).

## Declaration of interests

Dr. G. Dumas has received a lecture fee, as well as reimbursement of travel, from Baxter and Mundipharma. Dr. E. Canet has received lecturer and conference-speaker fees, as well as reimbursements of travel and accommodation expenses related to attending scientific meetings from Gilead. No other disclosures were reported.

## Appendix A Supplementary data

Supplemental appendix

Collaborators list
