# Supplementary material for: Outcomes, prognostic factors, and the role of intracranial pressure monitoring in severe community-acquired bacterial meningitis: a multicenter retrospective cohort study
Source: Lancet Reg Health Eur. 2026 Jul 6;68:101767. doi: 10.1016/j.lanepe.2026.101767 (PMC13355766; doi:10.1016/j.lanepe.2026.101767)
Supplement: Supplemental appendix [file mmc1.docx]

**Supplemental appendix**

**Authors:** Cindy Poirier, MD; Nicolas Terzi, MD, PhD; Nicolas Roesch, MD; Laurent Argaud, MD, PhD; Sarah Jouin, MD; Marc Le Pape, MD; Jean-Rémi Lavillegrand, MD, PhD; Hervé Hyvernat, MD; Romain Tymen, MD; Pierrick Lafarge, MD; David Bougon, MD; Pierrick Bauduin MD; Jérémie Mallet, MD; Julien Jabot, MD, PhD; Guillaume Navarro, MD; François Arrivé, MD; Juliette Bernier, MD; Michel Badet, MD; Maxens Decavele, MD, PhD; Anaïs Curtiaud, MD; Eddine Bendiab, MD; Baptiste Balanca, MD, PhD; Benoit Champigneulle, MD, PhD; Sami Hraiech, MD, PhD; Loïc Le Guennec, MD; Elodie Fournier, MD; Emmanuel Canet, MD, PhD; Romain Sonneville, MD, PhD; Guillaume Dumas, MD, PhD on behalf of the MAC-ICU Study Group.

**Content:**

[Table E1. Summary of evidence regarding invasive intracranial pressure monitoring in community-acquired bacterial meningitis 3](#_Toc227840630)

[Figure E1. Proportion of missing data in the dataset 4](#_Toc227840631)

[Missing data management 5](#_Toc227840632)

[Figure E2. Direct acyclic Graph illustrating the assumed causal structure between intracranial pressure (ICP) monitoring and 90-day functional outcome 6](#_Toc227840633)

[Figure E3. Flowchart of the study 6](#_Toc227840634)

[Figure E4. Distribution of the annual number of included patients over the study period 7](#_Toc227840635)

[Figure E5. Primary indications for endotracheal intubation 7](#_Toc227840636)

[Figure E6. Co-occurrence of diagnostic methods used to identify causative pathogens in community-acquired bacterial meningitis 8](#_Toc227840637)

[Figure E7. Antibiotic therapy received at ICU admission 9](#_Toc227840638)

[Analysis of invasive intracranial pressure monitoring use on the modified Rankin score at day 90 10](#_Toc227840639)

[Method 10](#_Toc227840640)

[Estimation of PS 10](#_Toc227840641)

[Overlap weights calculation 10](#_Toc227840642)

[Outcome analysis 10](#_Toc227840643)

[Analysis of the treatment effect 11](#_Toc227840644)

[Figure E8. Temporal trends in invasive intracranial pressure (ICP) monitoring use over the study period. 11](#_Toc227840645)

[Figure E9. Distribution of propensity scores according to invasive intracranial pressure monitoring 11](#_Toc227840646)

[11](#_Toc227840647)

[Figure E10. Covariate balance before and after overlap weighting 12](#_Toc227840648)

[Analysis of center effect 13](#_Toc227840649)

[Method 13](#_Toc227840650)

[Results 13](#_Toc227840651)

[Figure E11. Center-specific random intercepts before (Panel A, unadjusted model) and after (Panel B, adjusted multivariable model) adjustment for patient-level covariates. 14](#_Toc227840652)

[Post-hoc analysis 15](#_Toc227840653)

[Table E3. Alternative outcome thresholds and analytical approaches for the association between invasive ICP monitoring and functional outcome. 15](#_Toc227840654)

[References 16](#_Toc227840655)

**Table E1. Summary of evidence regarding invasive intracranial pressure monitoring in community-acquired bacterial meningitis**

| **Study, Author,  Year of publication** | **Design** | **Original  sample size** | **ICP monitoring vs no ICP monitoring** | **Devices used** | **Main results** | **Remarks** |
| --- | --- | --- | --- | --- | --- | --- |
| Lindvall et al., 2004^1^ | Retrospective, monocentric | 18 | 15 ICP / 3 no ICP | Intraparenchymal ICP monitors | Intracranial hypertension occurred in 93% Higher mean ICP and lower CPP were associated with mortality | Physiological association, No control group |
| Edberg et al., 2011^2^ | Retrospective, monocentric | 30 | 28 ICP / 2 no ICP | Ventricular catheters Intraparenchymal probes | ICP >20 mmHg in 27% CSF drainage frequently required mortality 20% | No control  group |
| Abulhasan et al., 2013^3^ | Retrospective, monocentric | 37 | 11 lumbar drain / 26 no drain | Lumbar CSF drainage | Lower mortality and better GOS  in lumbar drain group | Selection bias Non-randomized |
| Glimåker et al., 2014^4^ | Mixed  (control recruited retrospectively),  multicentric | 105 | 52 ICP-targeted care / 53 standard care | Mainly EVD | Lower mortality (10% vs 30%)  and better functional outcome in ICP-targeted group | Intervention–control design Intensive protocol |
| Muralidharan et al., 2014^5^ | Retrospective, monocentric | 39 | Mixed | ICP monitors | ICP monitoring associated with poor outcome | Likely confounding by indication |
| Larsen et al., 2017^6^ | Retrospective, monocentric | 39 | 39 ICP / 0 no ICP | Ventricular catheters Intraparenchymal probes | ICP elevation common Poor correlation with CT No clear outcome benefit | No control group |
| Wettervik TS et al., 2022^7^ | Retrospective, monocentric | 97 | 81 ICP / 16 no ICP | Mainly EVD | ICP insults common CSF drainage effective Outcome related to pressure reactivity index not ICP | Physiological association, No control group |
| Tetens et al., 2024^8^ | Retrospective, monocentric | 108 | 47 intracranial devices / 61 no device | Ventricular catheters Intraparenchymal probes | ICP elevation frequent CSF drainage often required Device-related complications occurred in 3 patients | Focus on feasibility and safety |
| Platz et al., 2025^9^ | Nationwide  population-based study | 638 | 305 regional routine use / 333 non-routine | Ventricular catheters Intraparenchymal probes | No difference in mortality or neurological sequelae | Indirect exposure definition |

CSF: cerebrospinal fluid; CT: computed tomography; CPP: cerebral perfusion pressure; EVD: External ventricular drainage; GOS: Glasgow Outcome Score; ICP: intracranial pressure

**Table E2.** **Structural, organizational, and neurosurgical characteristics of participating ICUs**

| **Characteristics** | **Centers**  **N=26**  **N(%) or Median [IQR]** |
| --- | --- |
| Number of ICU beds | 24.0 [18.5-25.0] |
| University-affiliated hospital | 24 (92.3) |
| **ICU type** |  |
| Medical-ICU | 19 (73.1) |
| Mixed-ICU | 4 (15.4) |
| Neuro-ICU | 3 (11.5) |
| Neurosurgeon available on site 24/7 | 17 (65.4) |
| Ease of access to the neurosurgeon (intensivist-rated, 0–10 scale)* | 8.0 [7.0-9.0] |
| Ease of communication with the neurosurgeon (intensivist-rated, 0–10 scale)* | 7.0 [6.0-8.0] |
| **Usual operator for ICP monitor insertion**  Neurosurgeon  Intensivist | 23 (92.0)  3 (8.0) |
| Number of ICU admissions during the study period, per center | 11,500 [7,875-13,875] |
| Number of CABM admissions to the ICU during the study period, per center | 75 [53-96] |
| Number of CABM patients requiring invasive mechanical ventilation during the study period, per center | 25.0 [13.0-39.0] |

Data are presented as medians and interquartile ranges [IQR], unless otherwise indicated.

*Centers were surveyed to provide a global assessment of the quality of their interactions with the neurosurgeon, based on intensivist-reported ratings.

ICU, Intensive care unit; CABM, community-acquired bacterial meningitis

**Figure E1. Proportion of missing data in the dataset**

Patients (n=704)

**Missing data management**

Missing data were handled using multiple imputation by chained equations (MICE), implemented with the mice package in R. Missing data were assumed to be missing at random.

The imputation model included all variables in the analytical model, the following auxiliary variables associated with missingness or outcome: vasopressor use on day 1, total Glasgow Coma Scale score, SOFA score, SOFA score minus Glasgow component, and indication for intubation. The outcome variable (mRS ≥3 at day 90) and study center were also included to preserve the association structure between predictors and outcome and to account for center-level clustering.

Thirty imputation datasets were generated (maxit = 3 iterations), exceeding the recommended threshold of having at least as many imputations as the percentage of incomplete cases^10^. Continuous variables were imputed using predictive mean matching (pmm) and binary variables using logistic regression (logreg). Results were pooled across imputed datasets using Rubin's rules.

**Figure E
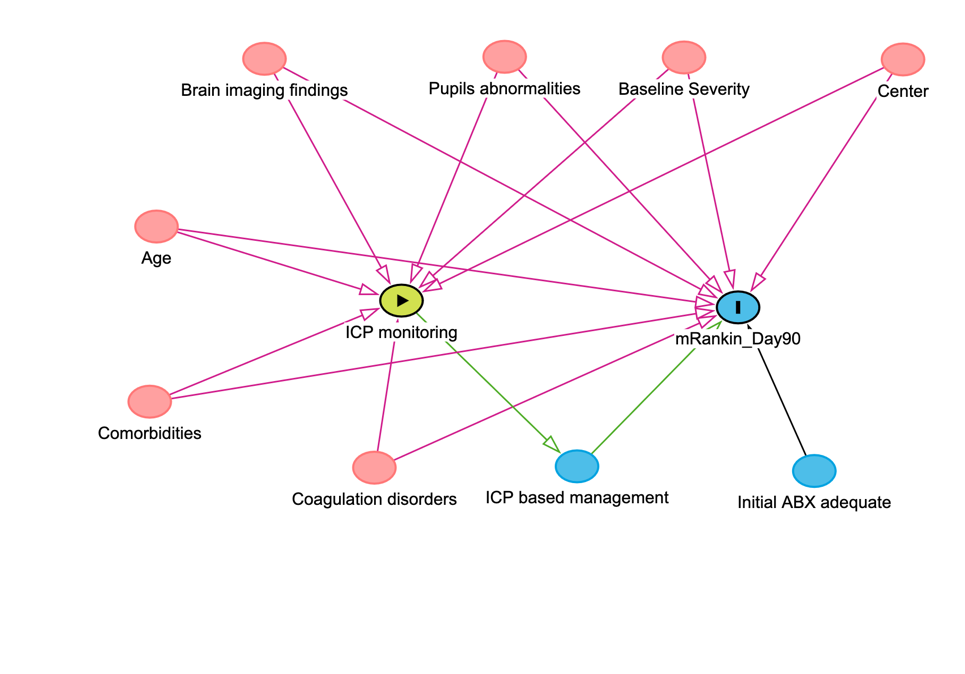
2. Direct acyclic Graph illustrating the assumed causal structure between intracranial pressure (ICP) monitoring and 90-day functional outcome**

Baseline characteristics, neurological severity, coagulation disorders, and center effects were considered confounders influencing both ICP monitoring and outcomes. ICP-based management was treated as a mediator and was not adjusted for. Adequate initial antibiotic therapy was modeled as an independent determinant of outcome. Figure drawn using <https://www.dagitty.net/>.

ICP: invasive cerebral pressure monitoring; ABX: antibiotics; mRankin_Day90: modified Rankin score at day 90 after ICU admission.

# **Figure E3. Flowchart of the study**

**
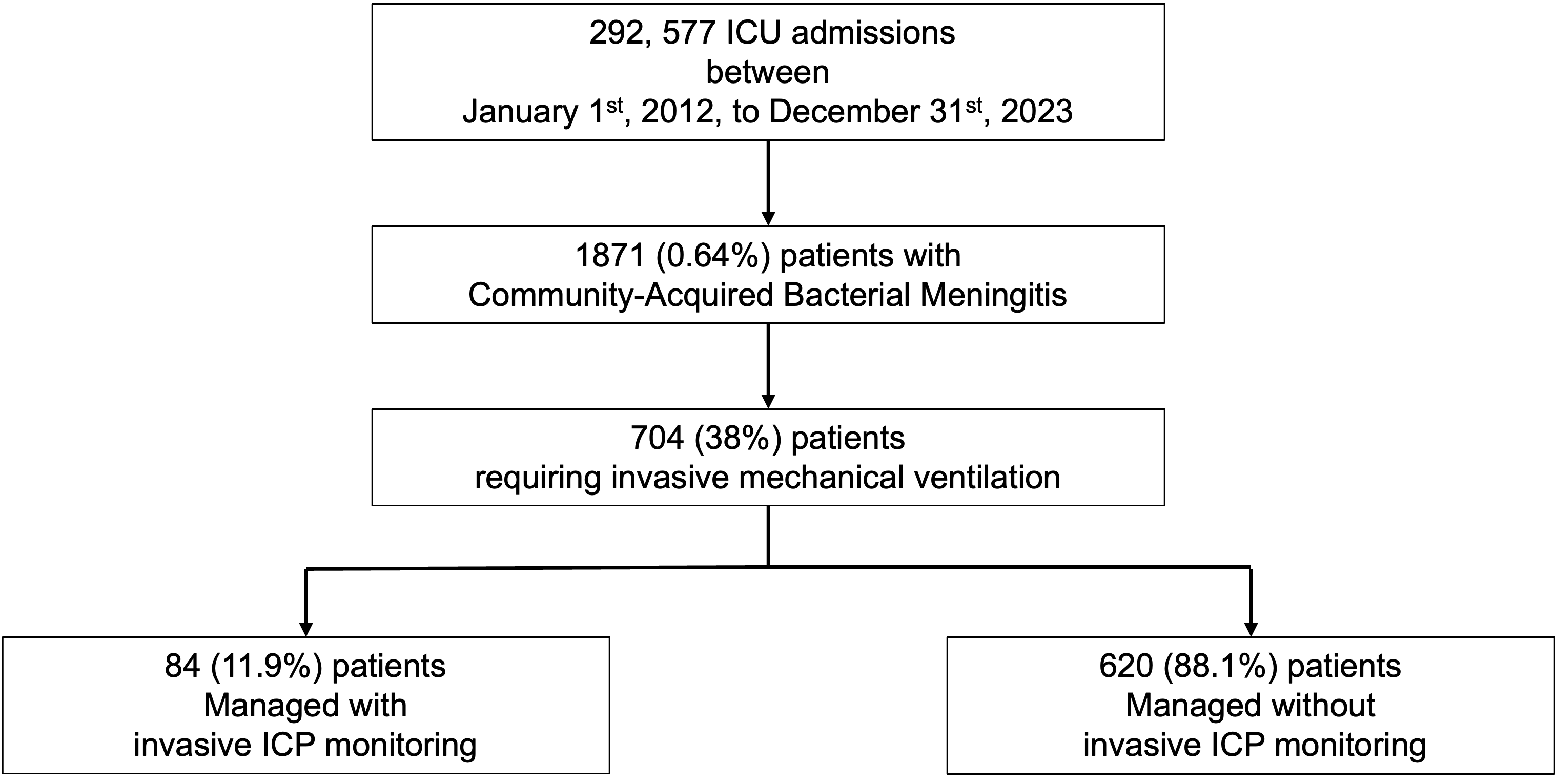
**

ICP: invasive cerebral pressure monitoring

**Figure E4. Distribution of the annual number of included patients over the study period**


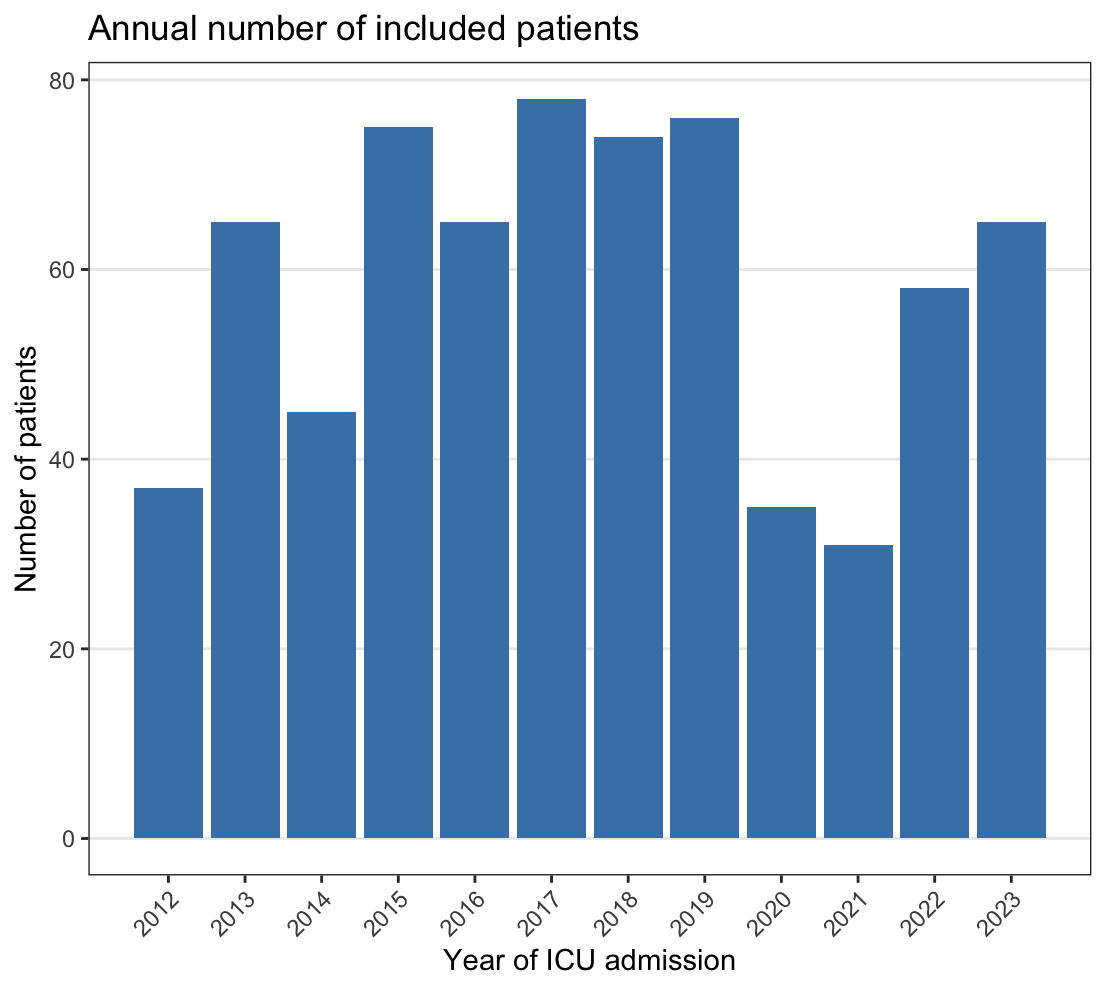


ICU: intensive care unit.

**Figure E5. Primary indications for endotracheal intubation**


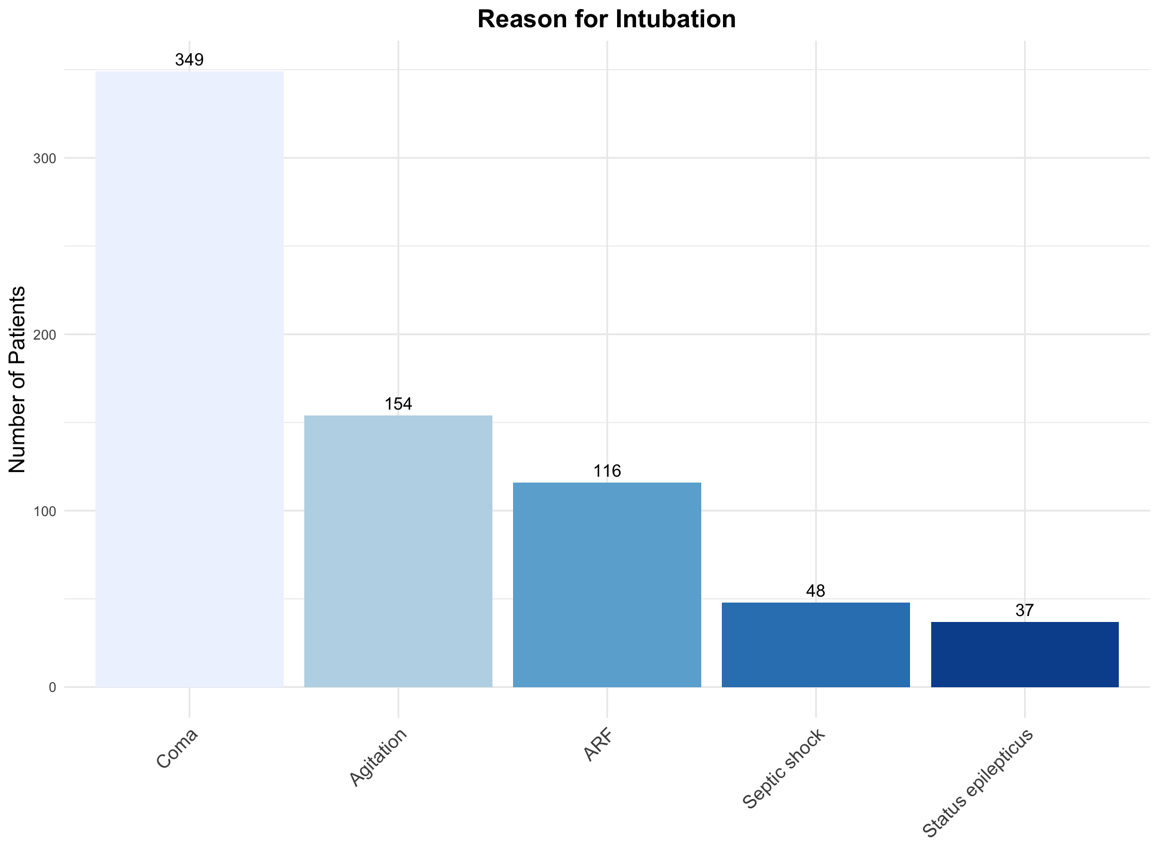


ARF: acute respiratory failure

**Figure E6. Co-occurrence of diagnostic methods used to identify causative pathogens in community-acquired bacterial meningitis**


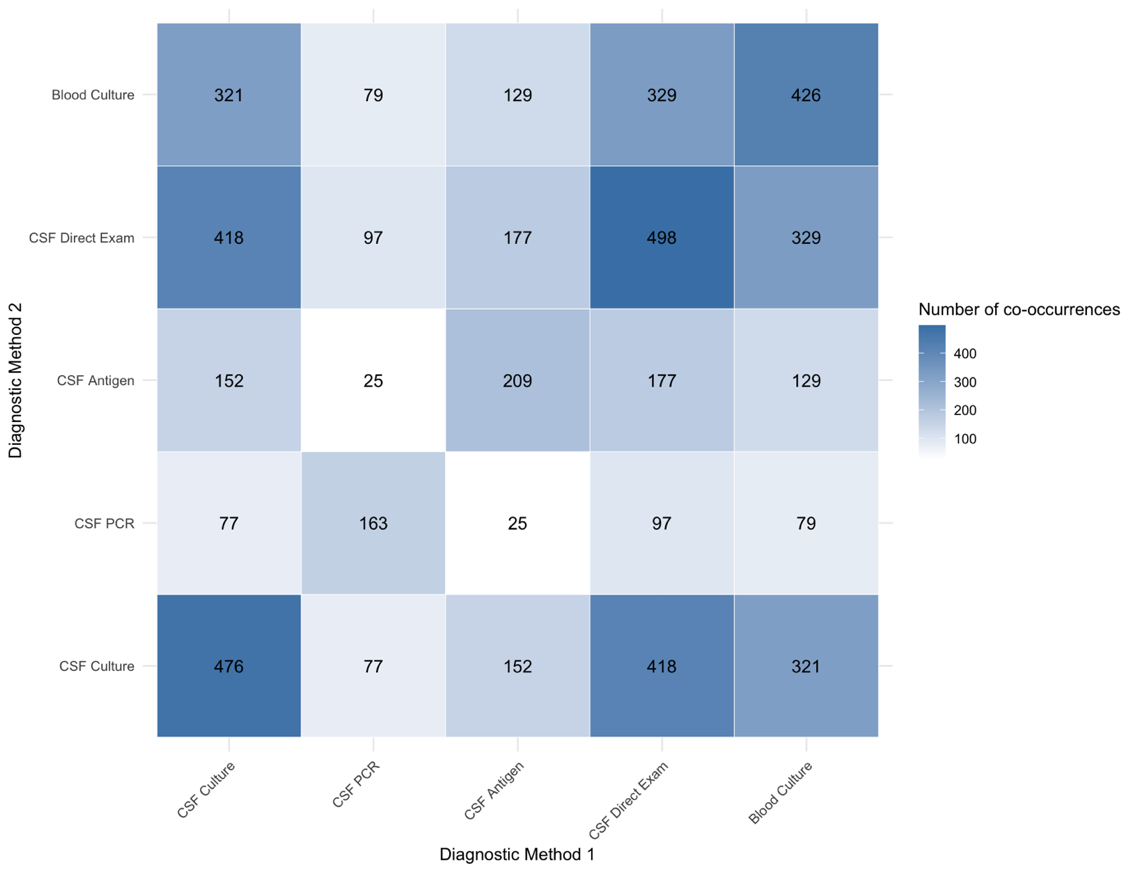


Heatmap displaying the number of patients in whom two diagnostic methods were used simultaneously for pathogen identification. Diagnostic methods include cerebrospinal fluid (CSF) culture, CSF polymerase chain reaction (PCR), CSF antigen testing, direct CSF examination, and blood cultures. Color intensity represents the number of co-occurrences.

CSF: cerebrospinal fluid; PCR: polymerase chain reaction

# **Figure E7. Antibiotic therapy received at ICU admission**


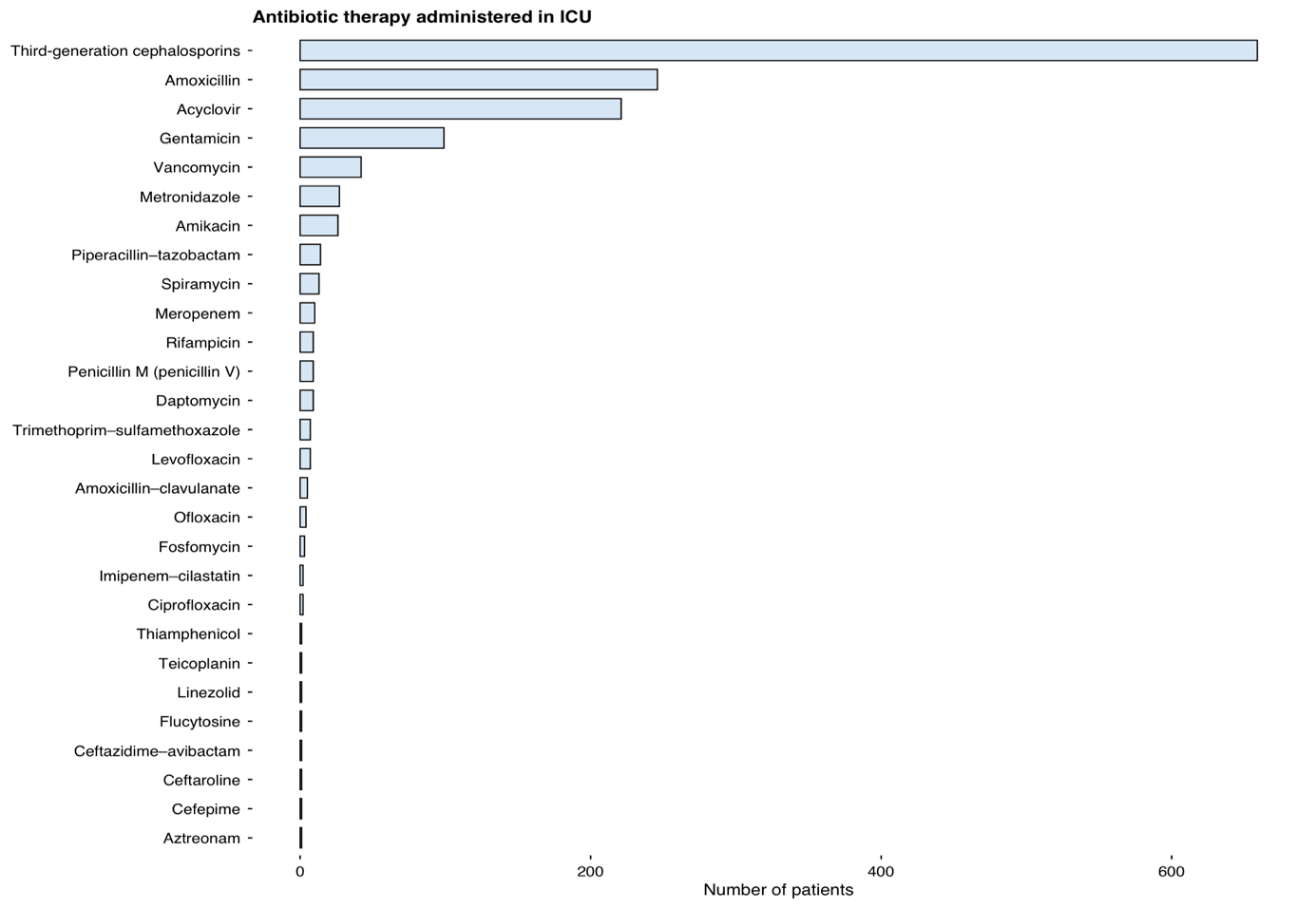


# **Analysis of invasive intracranial pressure monitoring use on the modified Rankin score at day 90**

## **Method**

Given the observational nature of the study, we used a propensity score–based overlap weighting approach to estimate the association between invasive intracranial pressure (ICP) monitoring and functional outcome at day 90, while accounting for confounding by indication. Overlap weighting is a causal inference method derived from the propensity score that emphasizes patients with a substantial probability of receiving either strategy, thereby mimicking the characteristics of a randomized clinical trial and improving covariate balance while limiting the influence of extreme weights^11,12^.

### *Estimation of PS*

The propensity score reflected each patient’s probability of receiving invasive ICP monitoring within the first 24 hours after ICU admission. It was estimated using a multivariable logistic regression model including prespecified clinically relevant confounders available at ICU admission: age, Charlson comorbidity index, Sequential Organ Failure Assessment score, Glasgow Coma Scale score, presence of pupillary abnormalities, coagulation disorders, abnormal brain imaging findings (cerebral edema, hydrocephalus, ischemic or hemorrhagic lesions), and the primary indication for endotracheal intubation. The study center was not included in the propensity score model but was accounted for in outcome models as a random effect.

Missing covariate data were handled using multiple imputation with chained equations, generating 30 imputed datasets ^10^. Propensity scores were estimated separately within each dataset and then averaged across imputations, as recommended to reduce bias and improve efficiency^13^. Different metrics were used to check whether the balance of confounders has been achieved, based on the comparison of these metrics before and after weighting^14^.

### *Overlap weights calculation*

Overlap weights were constructed as follows:

- patients who received ICP monitoring were weighted by 1-PS,
- whereas patients who did not receive ICP monitoring were weighted by PS,

where PS denotes the individual propensity score.

This approach assigns greater weight to patients with clinical equipoise and downweighs those with extreme probabilities of treatment assignment, thereby reducing variance and improving covariate balance without the need for weight truncation. Covariate balance before and after weighting was assessed using standardized mean differences. Absolute standardized differences below 10% were considered indicative of adequate balance ^15^.

### *Outcome analysis*

The primary endpoint was poor functional outcome at day 90, defined as a modified Rankin Scale score of 3-6. The association between invasive ICP monitoring and outcome was estimated using **weighted logistic regression models**, incorporating overlap weights and robust standard errors. The study center was included as a random effect to account for between-center variability. Effect estimates are reported as odds ratios (ORs) with 95% confidence intervals.

## **Analysis of the treatment effect**

# **Figure E8. Temporal trends in invasive intracranial pressure (ICP) monitoring use over the study period**


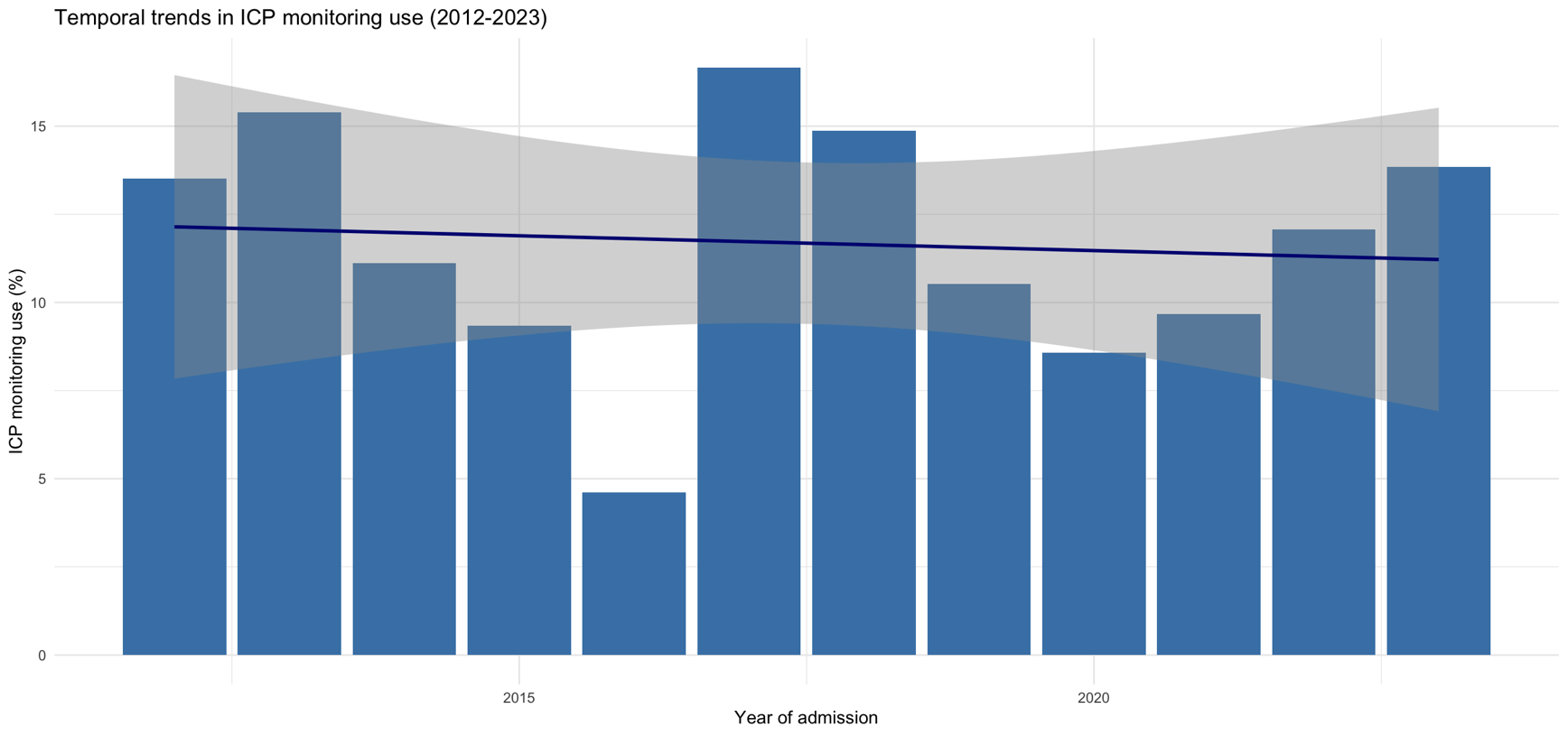


Bars represent the annual proportion of patients receiving invasive ICP monitoring. The solid line represents the linear trend over time with 95% confidence interval (shaded area).

No significant temporal trend was observed (chi-squared test for trend p=0.984), suggesting that ICP monitoring practices remained stable across the study period.

# **Figure E9. Distribution of propensity scores according to invasive intracranial pressure monitoring**


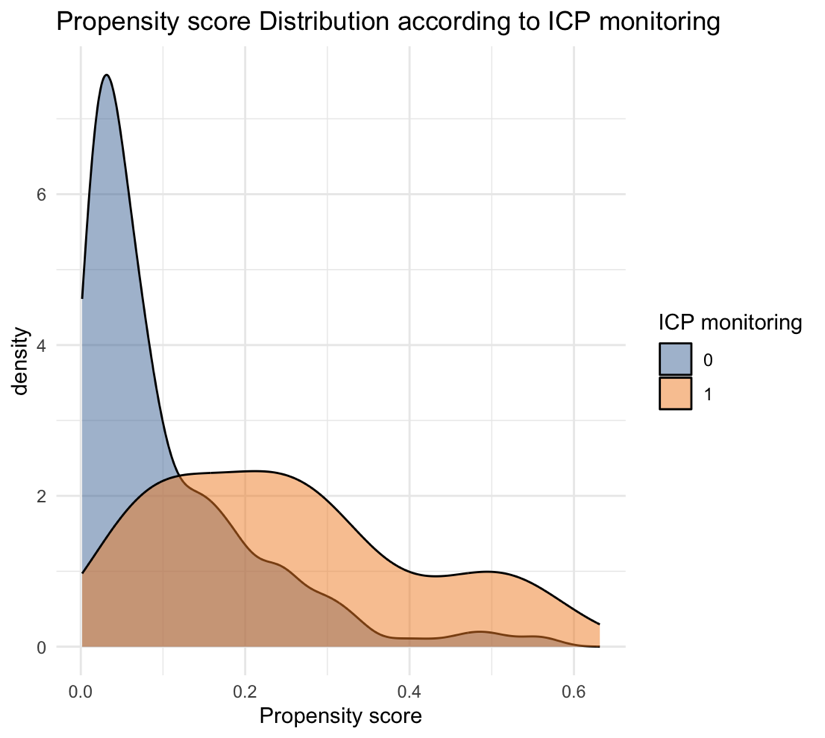


Kernel density plots show the distribution of estimated propensity scores for receiving invasive ICP monitoring among patients with (orange) and without (blue) ICP monitoring. The overlap between the two distributions indicates the region of common support, supporting the positivity assumption and justifying the use of overlap weighting for causal effect estimation.

ICP: invasive intracranial pressure monitoring

# **
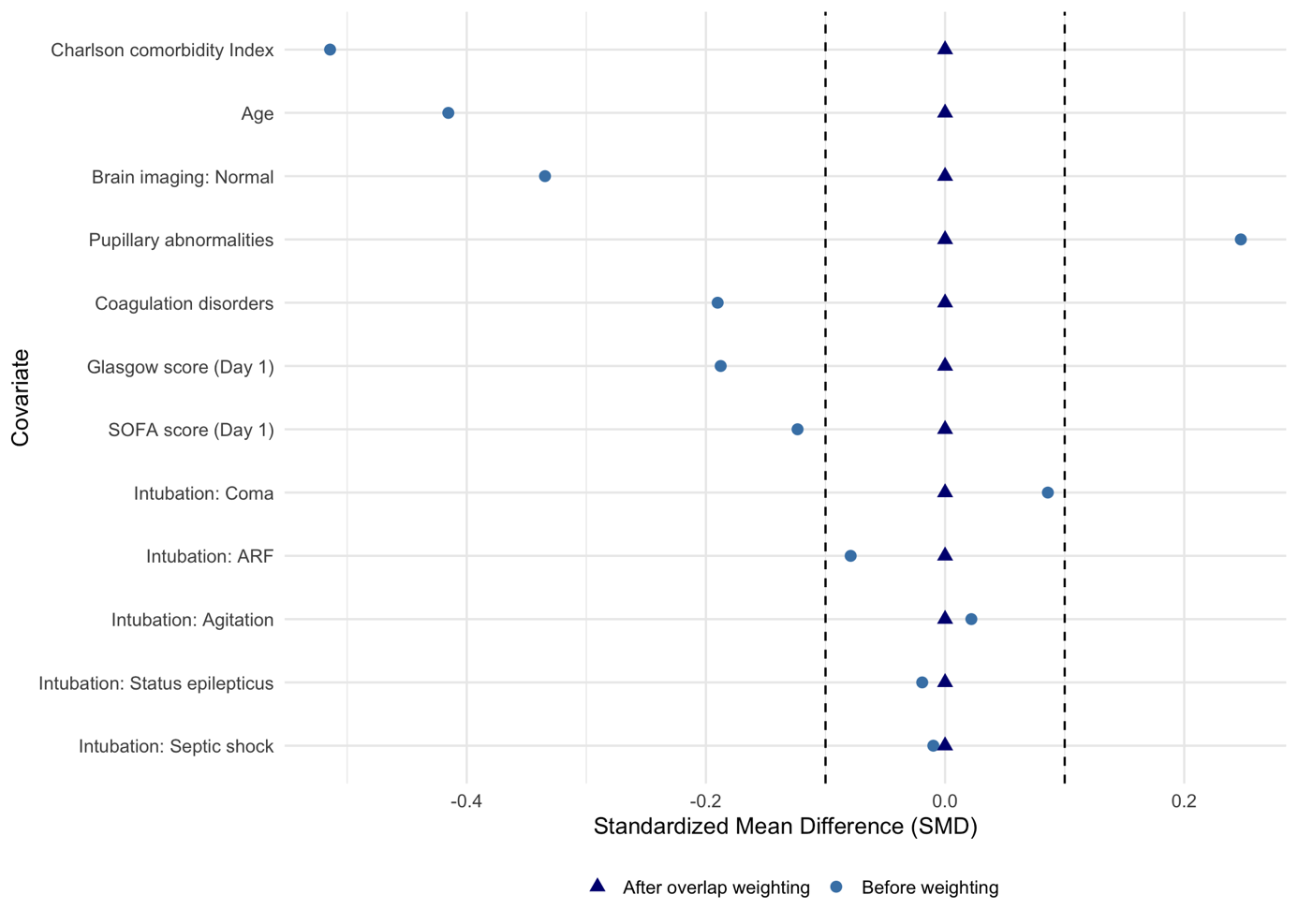
Figure E10. Covariate balance before and after overlap weighting**

Standardized mean differences for baseline covariates comparing patients with and without invasive intracranial pressure monitoring before weighting (light blue) and after overlap weighting (dark blue). Dashed vertical lines indicate the prespecified thresholds for acceptable imbalance.

# **Analysis of center effect**

## **Method**

Between-center variability in outcome risk was assessed using random intercept logistic regression models and quantified using the median odds ratio (MOR), calculated as exp(√2 × VarCorr(Centre) × Φ⁻¹(0.75)), where Φ⁻¹(0.75) is the 75th percentile of the standard normal distribution. Statistical significance of the between-center variance was assessed using a permutation test (1000 permutations)^16,17^.

To assess whether the effect of ICP monitoring varied across centers, a random slope model was fitted allowing both the intercept and the effect of ICP monitoring to vary by center.

The potential contribution of on-site neurosurgical availability to between-center heterogeneity was evaluated by including this variable as a fixed effect in separate logistic regression models.

## **Results**

The unadjusted MOR was 1.17 (permutation test p=0.164), suggesting modest and non-significant between-center variability in baseline outcome risk. After adjustment for patient-level covariates, the MOR increased to 1.42 (permutation test p=0.033), indicating residual between-center differences in management practices contributing to outcome variability beyond patient characteristics. Center-specific random intercepts are displayed in Supplemental Figure E10, showing that most centers clustered within one standard deviation of the mean, with no extreme outlying centers.

On-site neurosurgical availability was not significantly associated with functional outcome in unadjusted (OR: 1.16, p=0.481), adjusted (OR: 1.34, p=0.408), or fully adjusted models including ICP monitoring (OR: 1.34, p=0.412). The effect of ICP monitoring remained consistent after inclusion of neurosurgical availability (OR: 0.80, p=0.548).

A random slope model allowing the effect of ICP monitoring to vary by center showed negligible between-center variance in the treatment effect (SD=0.10), with model over-parameterization likely reflecting the limited number of ICP-monitored patients per center. These findings do not support meaningful heterogeneity in the effect of ICP monitoring across centers."

#
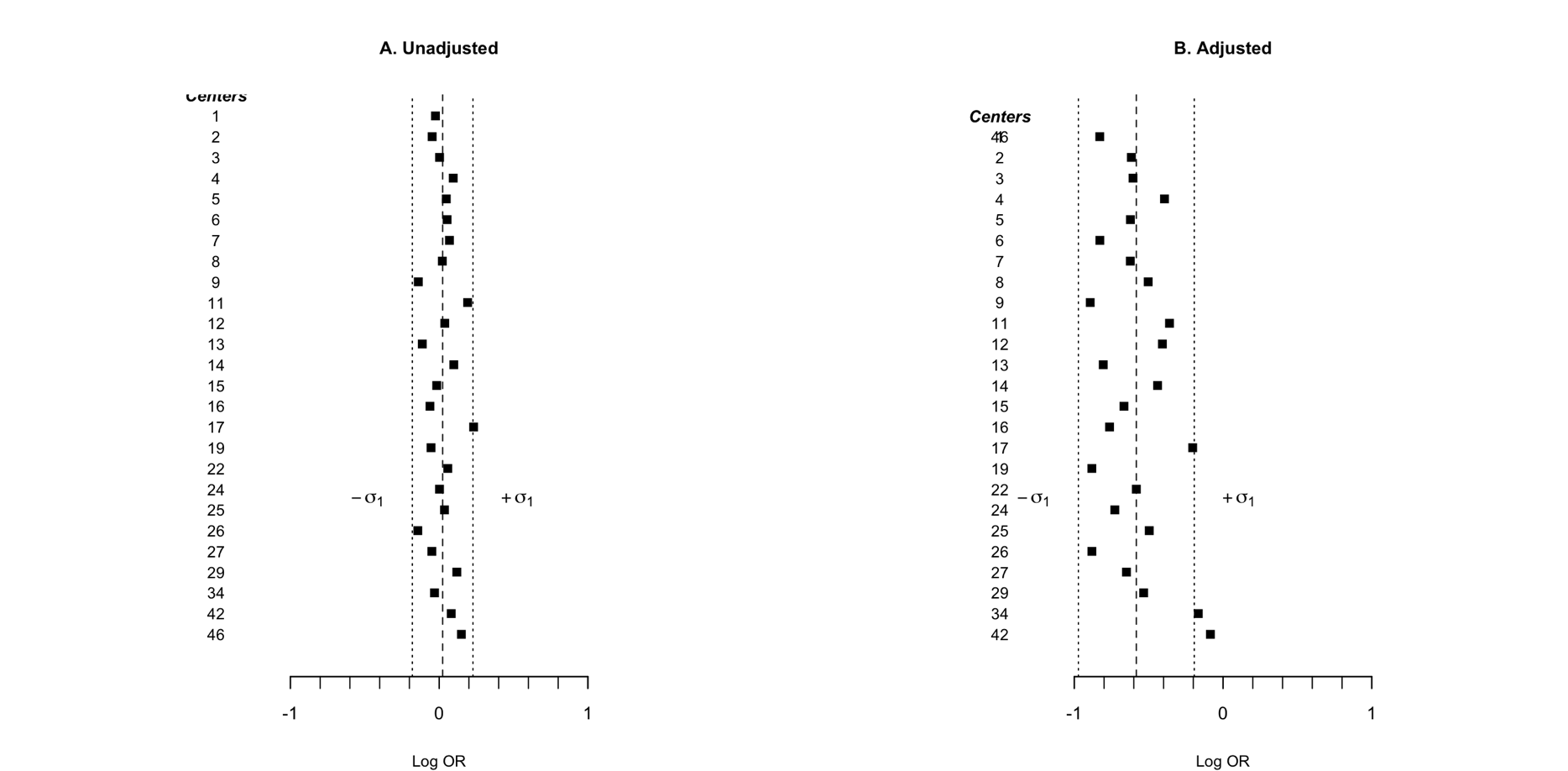
**Figure E11. Center-specific random intercepts before (Panel A, unadjusted model) and after (Panel B, adjusted multivariable model) adjustment for patient-level covariates**

Each square represents the center-specific log odds ratio for unfavorable functional outcome at day 90 relative to the overall mean (dashed vertical line). Dotted vertical lines represent ±1 standard deviation (±σ₁) of the between-center variance distribution.

# **Post-hoc analysis**

# **Table E3. Alternative outcome thresholds and analytical approaches for the association between invasive ICP monitoring and functional outcome**

| **Variable** | **Primary analysis (mRS ≥3)** | | **Sensitivity analysis (mRS ≥4)** | |
| --- | --- | --- | --- | --- |
|  | **OR (95% CI)** | **p-value** | **OR (95% CI)** | **p-value** |
|  | | | | |
| **Multivariable mixed-effects logistic regression** | | | | |
| Intracranial pressure monitoring (Yes) | 0·84 (0·45-1·58) | 0·589 | 0·92 (0·49-1·72) | 0·783 |
| Age (years) | 1·04 (1·03-1·05) | <0·001 | 1·03 (1·02-1·05) | <0·001 |
| Charlson Comorbidity Index (without age) | 1·04 (0·93-1·16) | 0·533 | 1·15 (1·03-1·29) | 0·014 |
| Associated septic shock (Yes) | 2·32 (1·47-3·65) | <0·001 | 2·00 (1·29-3·09) | 0·002 |
| Acute Kidney Injury (Yes) | 1·81 (1·18-2·78) | 0·007 | 1·92 (1·26-2·92) | 0·003 |
| Coagulation disorder (Yes) | 2·04 (1·35-3·08) | <0·001 | 2·09 (1·38-3·19) | <0·001 |
| Glasgow score Motor | 0·87 (0·79-0·97) | 0·012 | 0·83 (0·74-0·92) | <0·001 |
| Brain edema on imaging (Yes) | 2·22 (1·32-3·75) | 0·003 | 2·61 (1·56-4·36) | <0·001 |
| Hydrocephalus on imaging (Yes) | 3·36 (1·81-6·26) | <0·001 | 3·86 (2·11-7·05) | <0·001 |
| Ischemic lesions on imaging (Yes) | 2·94 (1·91-4·52) | <0·001 | 2·42 (1·59-3·69) | <0·001 |
| Hemorrhagic lesions on imaging (Yes) | 2·25 (1·19-4·27) | 0·013 | 1·91 (1·05-3·48) | 0·035 |
| Pupillary abnormalities (Yes) | 1·80 (1·10-2·95) | 0·020 | 1·66 (1·02-2·72) | 0·043 |
| Initial antibiotic therapy adapted (Yes) | 0·32 (0·16-0·62) | <0·001 | 0·45 (0·25-0·82) | 0·010 |
| Dexamethasone use (Yes) | 0·54 (0·35-0·82) | 0·005 | 0·51 (0·33-0·77) | 0·002 |
| Year of admission | 1·03 (0·98-1·09) | 0·267 | 1·03 (0·97-1·09) | 0·393 |
| **Propensity score-based overlap weighting (ICP monitoring vs. no ICP monitoring)** | | | | |
| Intracranial pressure monitoring (Yes) | 0·97 (0·48-1·96) | 0·931 | 1·08 (0·53-2·21) | 0·828 |
| **Ordinal logistic regression (mRS 0-6)** | | | | |
| Intracranial pressure monitoring (Yes) | 0·82 (0·42-1·59) | 0·549 | - | - |

Multivariable analysis based on mixed-effects logistic regression with center as random effect, pooled across 30 imputed datasets using Rubin’s rules.

Overlap weighting restricted to ICP monitoring inserted within 24 hours of ICU admission.

Ordinal regression fitted using cumulative link mixed models (clmm) with center as random effect, pooled across 30 imputed datasets.

*OR: odds ratio; CI: confidence interval; mRS: modified Rankin Scale*

# **References**

1 Lindvall P, Ahlm C, Ericsson M, Gothefors L, Naredi S, Koskinen L-OD. Reducing intracranial pressure may increase survival among patients with bacterial meningitis. *Clin Infect Dis Off Publ Infect Dis Soc Am* 2004; **38**: 384–90.

2 Edberg M, Furebring M, Sjölin J, Enblad P. Neurointensive care of patients with severe community-acquired meningitis. *Acta Anaesthesiol Scand* 2011; **55**: 732–9.

3 Abulhasan YB, Al-Jehani H, Valiquette M-A, *et al.* Lumbar drainage for the treatment of severe bacterial meningitis. *Neurocrit Care* 2013; **19**: 199–205.

4 Glimåker M, Johansson B, Halldorsdottir H, *et al.* Neuro-intensive treatment targeting intracranial hypertension improves outcome in severe bacterial meningitis: an intervention-control study. *PloS One* 2014; **9**: e91976.

5 Muralidharan R, Mateen FJ, Rabinstein AA. Outcome of fulminant bacterial meningitis in adult patients. *Eur J Neurol* 2014; **21**: 447–53.

6 Larsen L, Poulsen FR, Nielsen TH, Nordström C-H, Schulz MK, Andersen ÅB. Use of intracranial pressure monitoring in bacterial meningitis: a 10-year follow up on outcome and intracranial pressure versus head CT scans. *Infect Dis Lond Engl* 2017; **49**: 356–64.

7 Svedung Wettervik T, Howells T, Ljunghill Hedberg A, Lewén A, Enblad P. Intracranial pressure dynamics and cerebral vasomotor reactivity in community-acquired bacterial meningitis during neurointensive care. *J Neurosurg* 2022; **136**: 831–9.

8 Tetens MM, Roed C, Bodilsen J, *et al.* Use of intensive care, intracranial pressure monitoring, and external ventricular drainage devises in patients with bacterial meningitis, a cohort study. *Acta Neurochir (Wien)* 2024; **166**: 287.

9 Platz IL, Tetens MM, Andersen NS, *et al.* Mortality and sequelae associated with regional use of intracranial devices among patients with pneumococcal meningitis: a nationwide, population-based cohort study. *Clin Microbiol Infect* 2025; **31**: 979–86.

10 White IR, Royston P, Wood AM. Multiple imputation using chained equations: Issues and guidance for practice. *Stat Med* 2011; **30**: 377–99.

11 Austin PC, Stuart EA. Moving towards best practice when using inverse probability of treatment weighting (IPTW) using the propensity score to estimate causal treatment effects in observational studies. *Stat Med* 2015; **34**: 3661–79.

12 Boston 677 Huntington Avenue, Ma 02115 +1495‑1000. Causal Inference Book. Miguel Hernan. 2012; published online Oct 19. https://www.hsph.harvard.edu/miguel-hernan/causal-inference-book/ (accessed Nov 14, 2017).

13 Mitra R, Reiter JP. A comparison of two methods of estimating propensity scores after multiple imputation. *Stat Methods Med Res* 2016; **25**: 188–204.

14 Franklin JM, Rassen JA, Ackermann D, Bartels DB, Schneeweiss S. Metrics for covariate balance in cohort studies of causal effects. *Stat Med* 2014; **33**: 1685–99.

15 Ridgeway G, McCaffrey D, Morral A, Burgette L, Griffin BA. Toolkit for Weighting and Analysis of Nonequivalent Groups: A tutorial for the twang package. *R Vignette RAND* 2015. ftp://xyz.csail.mit.edu/pub/CRAN/web/packages/twang/vignettes/twang.pdf (accessed June 17, 2016).

16 Biard L, Darmon M, Lemiale V, *et al.* Center Effects in Hospital Mortality of Critically Ill Patients With Hematologic Malignancies. *Crit Care Med* 2019; **Online First**. DOI:10.1097/CCM.0000000000003717.

17 Dumas G, Demoule A, Mokart D, *et al.* Center effect in intubation risk in critically ill immunocompromised patients with acute hypoxemic respiratory failure. *Crit Care Lond Engl* 2019; **23**: 306.
