# Supplementary material for: Outcomes, prognostic factors, and the role of intracranial pressure monitoring in severe community-acquired bacterial meningitis: a multicenter retrospective cohort study
Source: Lancet Reg Health Eur. 2026 Jul 6;68:101767. doi: 10.1016/j.lanepe.2026.101767 (PMC13355766; doi:10.1016/j.lanepe.2026.101767)
Supplement: Collaborators list [file mmc2.docx]

| **First name** | **Surname** |
| --- | --- |
| François | Beloncle |
| François | Barbier |
| Nicolas | de Prost |
| Mathieu | Jozwiak |
| Cécile | Aubron |
| Frédéric | Pène |
| Damien | Du cheyron |
| Tai | Pham |
| Phan | Hoang |
| Florian | Contard |
| Clément | Brault |
| Rémi | Coudroy |
| Hafid | Ait Oufella |
| Ferhat | Meziani |
| Kada | Klouche |
